# New Species of the Purse-Web Spider Genus *Atypus* Latreille, 1804 from Southern China (Araneae, Atypidae), with the General Natural History of *Atypus* Spiders

**DOI:** 10.3390/insects16030301

**Published:** 2025-03-13

**Authors:** Yecheng Wu, Yang Liu, Zongguang Huang, Haiqiang Yin, Xiang Xu

**Affiliations:** College of Life Science, Hunan Normal University, Changsha 410081, China; ycwu1123@gmail.com (Y.W.); liu.y119@outlook.com (Y.L.); zghuang@hunnu.edu.cn (Z.H.)

**Keywords:** atypidae, COI, taxonomy, morphology, biological traits

## Abstract

Three species of the purse-web spider genus *Atypus* Latreille, 1804, collected from Hunan and Sichuan Provinces of China, are diagnosed and described as new to science: *A. yaozu* sp. nov. (♂♀), *A. siyiensis* sp. nov. (♂♀) and *A. yanjingensis* sp. nov. (♂♀). Detailed descriptions, photographs and DNA barcodes of the three new species and a distribution map of *Atypus* species in China are provided. Additionally, we enrich the general natural history of the genus *Atypus* through a decade of observation.

## Simple Summary

Purse-web spiders are fascinating creatures that build unique silk tubes for hunting and protection. They remain relatively unknown among non-professionals, primarily due to their living in distinctive yet inconspicuous webs. One end of each web remains underground, while the other end extends above the surface and maintains a nearly vertical orientation to the ground, blending seamlessly into the surrounding environment by attaching to plant stems or other nearby surfaces. Our understanding of the natural history of purse-web spiders was previously limited due to their underground habitat. However, through a decade of observation conducted both in the field and in the laboratory, we have significantly enriched our knowledge of their behaviors and habits, which we report in this study. Additionally, we describe three newly discovered purse-web spider species within the genus *Atypus*, collected from Hunan and Sichuan Provinces in China. We provide detailed descriptions, color photographs and DNA barcodes of all new species, and a distribution map of *Atypus* species in China.

## 1. Introduction

**Figure 2:**
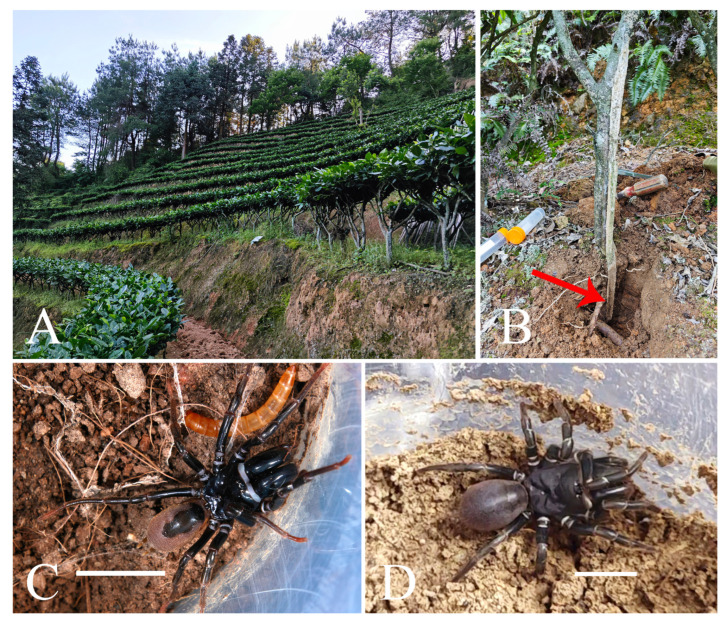
Microhabitat and general somatic morphology of *Atypus yaozu* sp. nov. (**A**,**B**) microhabitat; (**B**) purse-web (red arrow indicates the position of the spider); (**C**) male holotype (HNU1359); (**D**) female (HNU1360). Scale bars: (**C**,**D**) 5 mm.

Atypids are known for their burrowing behavior. Some of them live in underground burrows with silk-lined entrances (such as *Calommata* Lucas, 1837) (see figure 1 in Li et al. 2022 [1]), while others ingeniously extend their silken tubes and disguise an above-ground portion to ensnare unsuspecting insects (Figure 2B) (such as *Atypus* Latreille, 1804; *Sphodros* Walckenaer, 1835) [2,3,4].

Gertsch and Platnick [5] noted that J. T. Abbot first described the silken tubular webs of one spider species collected from America in 1792, coining the term “purse-web” (inspired by the fashionable slender purses of that era) to refer to the web, and consequently naming the spiders “purse-web spiders”. This species was later formally classified as *Sphodros abboti* Walckenaer, 1835 [5,6].

**Figure 3:**
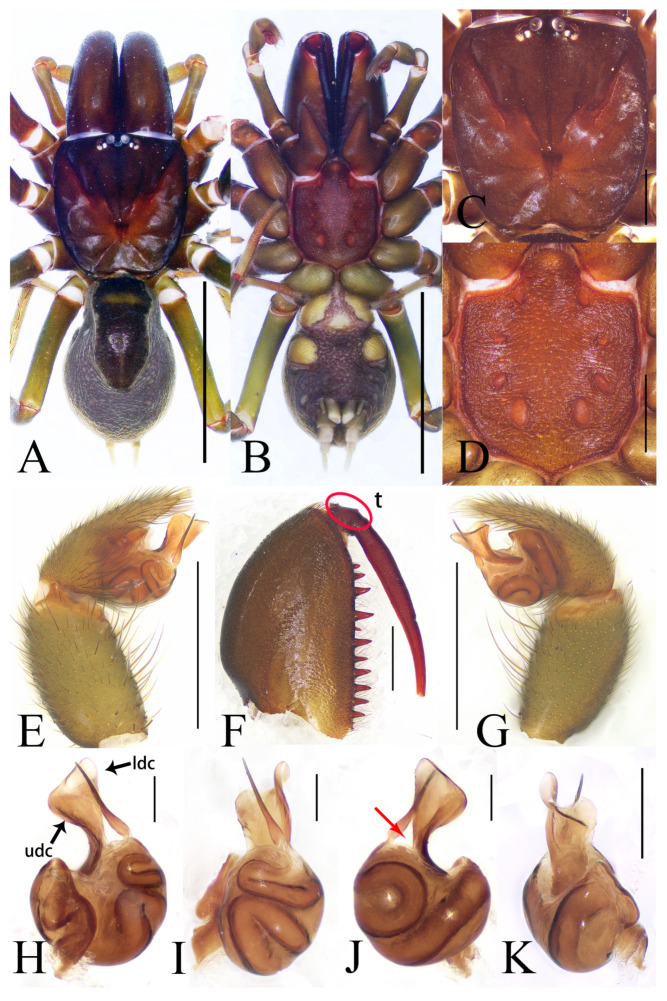
General somatic morphology and genital anatomy of *Atypus yaozu* sp. nov. male holotype (HNU1359). (**A**,**B**) habitus; (**A**) dorsal view; (**B**) ventral view; (**C**) carapace, dorsal view; (**D**) labium and sternum, ventral view; (**E**) left palpal, prolateral view; (**F**) left chelicera, inner-lateral view (“t” means tubercles); (**G**) left palpal, retrolateral view; (**H**) left palpal bulb, prolateral view (“udc” means upper distal corner, “ldc” means lower distal corner); (**I**) same, ventral view; (**J**) same, retrolateral view (red arrow indicates the wide triangular space between bases of embolus and conductor in lateral views); (**K**) same, dorsal view. Scale bars: (**A**,**B**) 5 mm; (**C**–**G**) 1 mm; (**H**–**K**) 0.2 mm.

The family Atypidae Thorell, 1870, as part of the infraorder Mygalomorphae, retains several typical plesiomorphic characteristics, such as the presence of a large tergite on the dorsum of the abdomen (Figure 2C,D) and six spinnerets (Figure 3B) [5]. The genus *Atypus* Latreille, 1804 is the type genus of the family (WSC 2025) [7]. Initially, the type species of *Atypus* was *Aranea subterranea* Roemer, 1789, but later this species was synonymized with *Atypus piceus* Sulzer, 1776 by Simon [8]. Before the establishment of the family Atypidae, *Atypus* was classified within the family Theraphosidae Thorell, 1869. Thorell [9] designated *Atypus* as the type genus of a separate subfamily (Atypinae of Theraphosidae), owing to its six spinnerets, a characteristic that distinguishes it from other theraphosids, especially those in the subfamily Theraphosinae Thorell, 1870 that possess only four spinnerets.

Bertkau [10] formally established the family Atypidae, initially comprising only the genus *Atypus*. Later, Thorell [11] divided the tribe Territelariae into five families, one of which was Calommatoidae Thorell, 1887 (originally comprising only the genus *Camptotarsus* Thorell, 1887, which Simon [12] considered a junior synonym of *Calommata*). Thorell also added *Atypus* and *Calommata* to Calommatoidae, thereby expanding the family to three genera at that time. Although Thorell also expressed that Calommatoidae should be considered as a junior synonym of Atypidae, he still proposed Calommatoidae as a separate family to reflect his disagreement with Bertkau’s earlier establishment of Atypidae, which had included only *Atypus*. In fact, they had different views in many aspects and had long debates, not just about Atypidae [13].

Two years later, based on specimens from a broader range, Simon [12] questioned Thorell’s conclusions and revised the classification by dividing Atypidae into three subfamilies (Brachybothriinae, Hexurinae, Atypinae) and placing *Atypus* and *Calommata* within the subfamily Atypinae. Simon [14] further refined Atypidae to include only the genera *Atypus* and *Calommata*, defining the family by several key characteristics: (1) the maxillary lobe (straight and conical in *Atypus* or arched outward in *Calommata*) occupying the entire length of the coxa’s anterior border (see figures 139–140 in Simon 1892 [12]); (2) a sternum with four pairs of sigilla, with the posterior pair being large and closely positioned, and the remaining pairs decreasing in size from back to front, while the first pair remains distinct from the groove of the labial piece (if the groove present) (Figure 3D); and (3) a complex male bulb with an apical point and conductor (see figure 135 in Simon 1892 [12]).

Kraus and Baur [15] observed the existence of two distinct types of spermathecae and palps among species formerly classified under *Atypus*, and they proposed that only the Palaearctic species should remain within *Atypus*, while *Sphodros* (originally established by Walckenaer in 1835 [6], with *Sphodros abboti* as its type species and later transferred to *Atypus* by McCook in 1888 [16]) should be recovered for the species that are exclusively found in the Nearctic region.

The current classification of the family Atypidae follows the definition by Gertsch and Platnick [5], who removed *Sphodros* from the synonymies of *Atypus*. Schwendinger [2] revised the genus *Atypus* globally, including four species from China. Zhu et al. [17] focused on revising *Atypus* species in China, providing descriptions of 13 species from the country, including seven new to science, and discussing the zoogeography of the genus. Li et al. [18] described two new species from Hainan Island of China and provided clear digital photographs of the female genitalia and male palp. Since then, no new *Atypus* species have been described globally.

The family Atypidae consists of 56 known species classified into three genera: *Atypus*, *Calommata* and *Sphodros*. In comparison to *Calommata* and *Sphodros*, *Atypus* dominates the family Atypidae in terms of species diversity, boasting 33 known species, which exceeds the combined total of 23 species found in the other two genera. Furthermore, within China, *Atypus* is the most speciose genus, with 15 species discovered, alongside only five known *Calommata* species [2,7,17,18,19,20].

*Atypus* species resides primarily in the underground portion of their purse-webs. Almost all behaviors, such as predation, mating and egg-laying, take place entirely within the web (both underground and above-ground portions). This makes it significantly challenging to observe and understand their specifics, as these activities never take place outside the webs.

The purse-webs provide a concealed environment that protects the spiders from predators and environmental disturbances. As a result, scientists have limited opportunities to witness and study the various behaviors of *Atypus* species in their natural setting. This lack of accessibility has historically posed a substantial challenge in gaining a comprehensive understanding of their activities and behaviors.

Although our understanding of the natural history of atypids is limited due to their secretive lifestyle, it has been confirmed that *Atypus* spiders are perennial spiders, with females potentially living for many years, up to 8–10 years according to Schwendinger [2]. The early literature, though scarce, also provide some insights into their general natural history [2,5,10,12].

In this study, we present a detailed taxonomic description of three newly discovered *Atypus* species from southern China. DNA barcodes derived from the mitochondrial cytochrome c oxidase subunit I (COI) gene of the new species are provided. Additionally, through a decade of field and laboratory observations, we offer an enriched understanding of the general natural history of the genus *Atypus*, complemented by detailed illustrations depicting various aspects such as weaving behavior, defecation behavior, and more.

## 2. Materials and Methods

### 2.1. Specimens Examination

**Figure 1:**
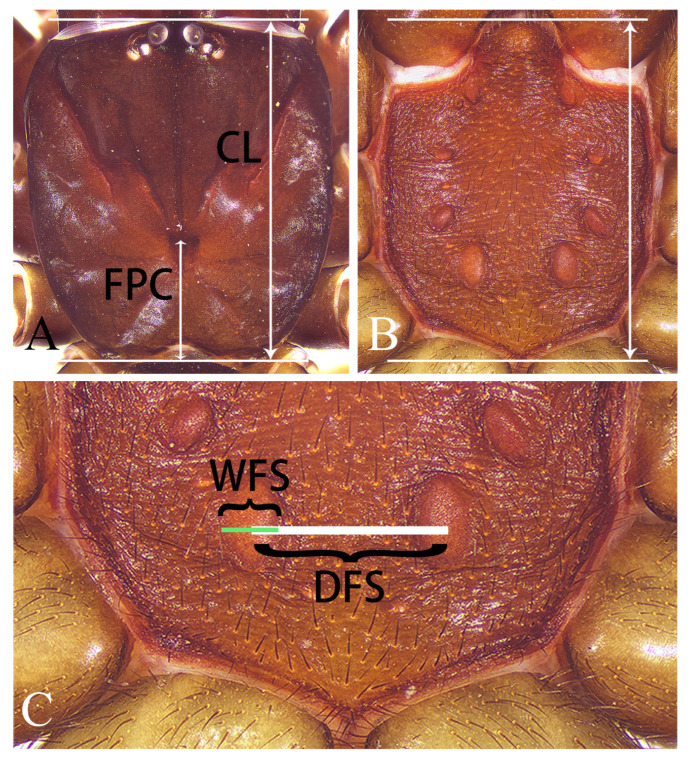
Illustration of selected measurement methods. (**A**) illustration of R1, the ratio of the distance between the fovea and the posterior end of the carapace (FPC) to the total length of the carapace (CL); (**B**) illustration of the sternum length measurement (including the fused labium); (**C**) illustration of R2, the ratio of the distance between the centers of the fourth pair of sigilla (DFS) to the width of the fourth sigilla (WFS).

**Figure 4:**
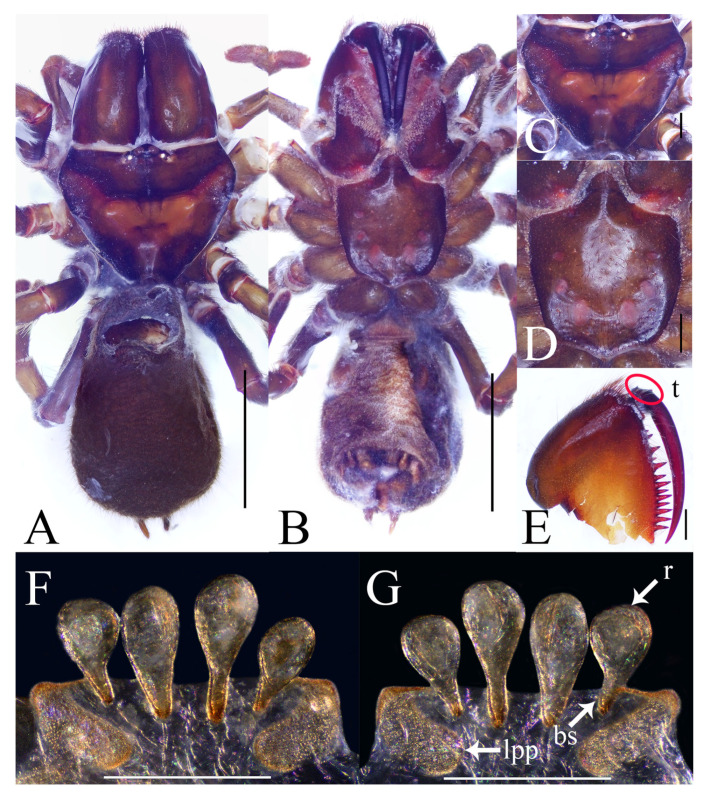
General somatic morphology and genital anatomy of *Atypus yaozu* sp. nov. female (HNU1360). (**A**,**B**) habitus; (**A**) dorsal view; (**B**) ventral view; (**C**) carapace, dorsal view; (**D**) labium and sternum, ventral view; (**E**) left chelicera, inner-lateral view (“t” means tubercles); (**F**) vulva, ventral view; (**G**) same, dorsal view (“r” means receptaculum, “bs” means basal stalk, “lpp” means lateral pore patch). Scale bars: (**A**,**B**) 5 mm; (**C**–**E**) 1 mm; (**F**,**G**) 0.5 mm.

All specimens were excavated from their subterranean silk tubes. They were collected alive and stored in absolute ethanol. The right leg I was removed for subsequent DNA extraction and the remains were preserved in 75% ethanol for morphological examination. Specimens were examined under an Olympus SZX16 stereomicroscope (Olympus Corporation, Tokyo, Japan) and an Olympus BX53 compound microscope (Olympus Corporation, Tokyo, Japan). After being dissected from the body, chelicerae of both sexes, male palp, and female genitalia (cleared with pancreatin by incubation at 37 °C overnight) were photographed using a Canon 80D camera (Canon Inc., Tokyo, Japan) mounted on an Olympus BX53 compound microscope (Olympus Corporation, Tokyo, Japan). All ecological photographs were taken with a Canon EOS R7 (Canon Inc., Tokyo, Japan). The other photos were photographed with a Qianjiayi industrial camera (Shenzhen Qianjiayi Technology Co., Ltd., Shenzhen, China). All morphological measurements were calculated using a LEICA M205C stereomicroscope (Leica Microsystems, Wetzlar, Germany) and given in millimeters. Eye diameters are taken at the widest point. Leg segments are measured on their dorsal sides. The length of the sternum is measured from the anterior margin of the fused labium to the posterior end of the sternum (as indicated by the white double-headed arrow in Figure 1B), and the width is taken at its widest point. The total length is measured including the chelicerae. Illustration of several key structures are provided: the upper distal corner and lower distal corner of the palpal organ are shown in Figure 3H; the lateral pore patch, receptaculum and basal stalk of the vulva are depicted in Figure 4G; tubercles on the fang of chelicera are illustrated in both Figure 3F and Figure 4E. Based on an extensive literature review and specimen examination, we propose that the following characteristics are also effective in taxonomic identification: the ratio of the distance between the fovea and the posterior end of the carapace (FPC) to the total length of the carapace (CL), abbreviated as FPC/CL = R1 (Figure 1A), the length-to-width ratio of the sternum (illustrated in Figure 1B), and the sizes, shapes, and relative positions of sigilla. Additionally, we introduce a new diagnostic feature: the ratio of the distance between the fourth pair of sigilla (DFS) to the width of the fourth sigilla (WFS), abbreviated as DFS/WFS = R2 (Figure 1C). DFS is specifically measured as the distance between the centers of the two sigilla, whereas WFS is measured at the broadest point of the sigilla (as shown in Figure 1C). It should be noted that all the aforementioned ratios are approximate estimates. All specimens examined in this study are deposited in the College of Life Sciences, Hunan Normal University (HNU), China.

### 2.2. Molecular Analysis

The genomic DNA was extracted from the leg tissue of adult spiders, employing the TlANamp Genomic DNA Kit from TianGen Biotech (Beijing, China) Co., Ltd., strictly following the manufacturer’s protocol (https://en.tiangen.com/content/details_43_4224.html, accessed on 1 October 2024). A fragment of the mitochondrial cytochrome c oxidase subunit I (COI) gene was amplified for 16 samples representing three new species, using the primer pairs: LCO1490 (5′-GGTCAACAAATCATAAAGATATTGG-3′) [21] and HCO2198 (5′-TAAACTTCAGGGTGACCAAAAAATCA-3′) [21]. Subsequently, the PCR products were purified and sequenced by Sangon Biotech (Shanghai, China) Co., Ltd. PCR reaction protocol and sequence data inspection follow Wheeler et al. [22]. Sequences were verified using BLAST (https://www.ncbi.nlm.nih.gov, accessed on 10 October 2024) and are deposited in GenBank. GenBank accession numbers for samples provided in this study are listed in Table S1.

The genetic distance of the COI gene was calculated using MEGA version 11 [23] with the following parameters: 1000 bootstrap replications based on both *p*-distance and Kimura 2-parameter (K2P) substitution models respectively, and other parameters were set the default. We compiled two datasets, Dataset I and Dataset II, to run the genetic distance analyses for calculating the interspecific and intraspecific genetic distance, respectively. All sequences used in the analyses were derived from our newly obtained data and the NCBI public database (https://www.ncbi.nlm.nih.gov, accessed on 10 October 2024).

### 2.3. Natural History Observation

Most behavioral and natural history images of *Atypus* spiders were captured using a Canon EOS R7 (Canon Inc., Tokyo, Japan), while a small number of images documenting nocturnal activities were taken with a SONY FDR-AX60 (Sony Corporation, Tokyo, Japan) utilizing its night vision function. Photographs inside the purse-web were taken using a DY8-M5-V26 industrial endoscope (Shenzhen Daying Technology Co., Ltd., Shenzhen, China). The specific behavioral details were recorded through long-term observations, while the timing and duration of various life-history stages were documented based on extended monitoring and statistical records. The timings reported in this study represent general patterns derived from our field collections in China (primarily in southern China) and laboratory observations.

We expand our understanding of their activities through field and laboratory observations, particularly during periods in the laboratory when their old webs have been destroyed and their newly constructed webs have not yet thickened, enabling us to observe some of their behaviors through the tubular web.

## 3. Results

### 3.1. Taxonomy

Family Atypidae Thorell, 1870

Genus *Atypus* Latreille, 1804

Type species. *Aranea subterranea* Roemer, 1789; synonym of *Atypus piceus* (Sulzer, 1776)

Diagnosis: It is worth noting that males of only *Atypus* in Atypidae possess marginal ridges of the sternum, a trait unique to this genus (Figure 3D) [5].

*Atypus* resembles *Sphodros* by having the labium fused to the sternum (Figure 1B) and possessing tubercles on the base of the fangs (Figure 3F and Figure 4E) but can be distinguished by the presence of a short, straight, spike-like embolus, and a short, straight and distally widened conductor in males (Figure 3E,G–K) (vs. the presence of a long, curved embolus and a long, curved conductor in *Sphodros*) (see figures 25–26 in Gertsch and Platnick 1980 [5]); and by the presence of two lateral pore patches, and bulbous or pyriform receptacula with their bases separated in females (Figure 4F,G) (vs. without pore patches, but with four highly coiled receptacula and their bases fused in pairs) (see figures 29, 30 in Gertsch and Platnick 1980 [5]).

*Atypus* also resembles *Calommata* by having a straight, spike-like embolus and a straight, broad, lobular conductor in males [5] but differs by the distally widened conductor, an almost rounded palpal bulb and an acuminate palpal cymbium in males (Figure 3E,G–K) (vs. a slender conductor, irregular palpal bulb and truncated palpal cymbium in *Calommata*) (see figure 6f in Li et al. 2022 [1]); and by receptacula without distinct granules in females (Figure 4) (vs. receptacula with granules, overall like cauliflowers in *Calommata* (see figure 7f–g in Li et al. 2022 [1]).

*Atypus yaozu* sp. nov. (瑶族地蛛)

urn:lsid:zoobank.org:act:38AD98C3-1F04-455E-B7CD-8F3C7F98B71C

Figure 2, Figure 3 and Figure 4

Type material. Holotype male (HNU1359): CHINA: Hunan Province, Chenzhou City, Yizhang County, Mangshan Town, Yaozu Village, 25.00° N, 112.83° E, 127 m a.s.l., 14 May 2024, YC Wu, Y. Liu, SX Zhu, W. Zhu, JM Fan leg.

Paratype: One female (HNU1360), same data as for holotype.

Etymology. The specific name is a noun in apposition and refers to the type locality, a village inhabited by the Yao ethnic minority.

Diagnosis: The male of this new species resembles that of *A. tibetensis* Zhu et al. 2006 in the shape of the palpal bulb (cf. Figure 3E,G–K with Zhu et al. 2006: figures 108–110 [17]) but can be distinguished by the presence of a wide triangular space between the embolus and conductor in lateral views (Figure 3H–K), the first pair of sigilla being obviously larger than the second, and the fourth pair being oval and oriented vertically (Figure 3D) (vs. no apparent space between the embolus and conductor in lateral views, the first and second pairs of sigilla almost equal in size, and the fourth pair nearly top-shaped and inclined in *A. tibetensis*) (see figures 107–110 in Zhu et al. 2006 [17]). It also resembles *A. baotingensis* Li et al. 2018 in the shape of the palpal bulb (cf. Figure 3E,G–K with Li et al. 2018: figure 2G–I [18]) but can be distinguished by a shorter embolus, whereas in *A. baotingensis*, the embolus longer and distinctly extends beyond the conductor.

Female of the new species resembles that of *A. jianfengensis* Li et al. 2018 in the shape of the receptacula (cf. Figure 4F,G with Li et al. 2018: figure 4D–E [18]) but can be distinguished by the rectangular pore patches with rounded corners and the third pair of sigilla being oval (Figure 4D,F,G) (vs. large, rounded pore patches and teardrop-shaped third sigilla in *A. jianfengensis*) (see figure 4B, D–E in Li et al. 2018 [18]). It also resembles that of *A. largosaccatus* Zhu et al. 2006 in the shape of the receptacula (cf. Figure 4F,G with Zhu et al. 2006: figures 42–43 [17]) but can be distinguished by the pore patches being rectangular with rounded corners, the median pair of receptacula larger than the lateral pair, and the first pair of sigilla positioned close to the anterior margin of the sternum (Figure 4D,F,G) (vs. small, rounded pore patches, receptacula equal in size, and the first pair of sigilla remote from the anterior sternal margin in *A. largosaccatus*) (see figures 41–43 in Zhu et al. 2006 [17]).

Description. Male (holotype). TL 10.70. CL 3.93, CW 3.57, AL 4.52, AW 2.96, FPC 1.50. Carapace brown (Figure 3C). Fovea transverse, occupying about 1/10 of carapace width at the same horizontal level, R1 0.38×. Radial grooves distinct (Figure 3C). Eye diameter and inter-distances: AME 0.25, ALE 0.21, PME 0.16, PLE 0.19; AME–AME 0.17, AME–ALE 0.11, PME–PME 0.62, PME–PLE 0.04. MOA 0.37, front width 0.64, back width 0.83. Labium wider than long (Figure 3D). Sternum reddish brown, 3.11 long, 2.26 wide, moderately rough, and clothed with fine black hairs. Sigilla deeply imprinted, DFS 0.82, WFS 0.18, with the second pair irregular and smallest, others oval, R2 4.5× (Figure 3D). Chelicerae brown, with 13 teeth on promargin arranged in a single row, the basal two fairly small and the fifth distal one noticeably smaller than the others (Figure 3F).

Abdomen dark brown, pyriform, with the dorsal scutum gloss black occupying about 7/10 of abdomen length (Figure 3A,B). Six spinnerets: ALS 0.32 long, PMS 0.56 long, four-segmented PLS with lengths as follows: basal 0.30, median 0.38, subapical 0.23, apical 0.40, total 1.31.

Legs slender, greenish brown, with coxae and trochanters reddish brown (Figure 2C). Spines present on all metatarsi; metatarsus IV with 16 ventral spines. Leg formula: 1423. Leg lengths are provided in Table 1.

Male palp (Figure 3E,G–K): upper corner small, lower distal corner triangular, distal margin of conductor strongly curved; embolus thin, with a wide triangular space between bases of itself and conductor in lateral views.

Female (paratype). TL 17.38. CL 4.96, CW 6.29, AL 8.75, AW 5.14, FPC 2.03. Carapace reddish brown (Figure 4C). Fovea transverse, occupying about 2/9 of the carapace width at the same horizontal level, R1 0.41×. Eye diameter and inter-distances: AME 0.35, ALE 0.27, PME 0.20, PLE 0.23; AME–AME 0.19, AME–ALE 0.21, PME–PME 0.97, PME–PLE 0.04. MOA 0.45 long, front width 0.88, back width 1.50. Labium wider than long (Figure 4D). Sternum reddish brown, 4.96 long, 3.95 wide, smooth, and clothed with fine black hairs. Sigilla slightly concave, DFS 1.28, WFS 0.49, with the first pair irregular in shape, the fourth pair oval and separated from each other by their longitudinal diameter; R2 2.6× (Figure 4D). Chelicerae reddish brown, with 13 teeth on promargin in a single row and the first and fourth ones extremely small (Figure 4E).

Abdomen pyriform and dark brown (Figure 4A,B). Six spinnerets: ALS 0.53 long, PMS 0.69 long, four-segmented PLS with lengths as follows: basal 0.62, median 0.53, subapical 0.50, apical 0.91, total 2.56.

Legs stronger than those of the male, reddish brown (Figure 2D). Spines present on all metatarsi; metatarsus IV with 13 ventral spines. Leg formula: 4123. Leg lengths are provided in Table 2.

Vulva (Figure 4F,G): Two lateral pore patches rectangular, with rounded corners; receptacula pyriform, with obvious basal stalks and the median pair larger than the lateral pair.

Habitat. Purse-webs were found attached to tea plants.

Distribution. Known only from the type locality, Yaozu Village (Yizhang County, Hunan Province, China).

*Atypus siyiensis* sp. nov. (泗义地蛛)

urn:lsid:zoobank.org:act:1F093142-6AB5-42D6-A56C-4AFF284FD6F0

**Figure 5:**
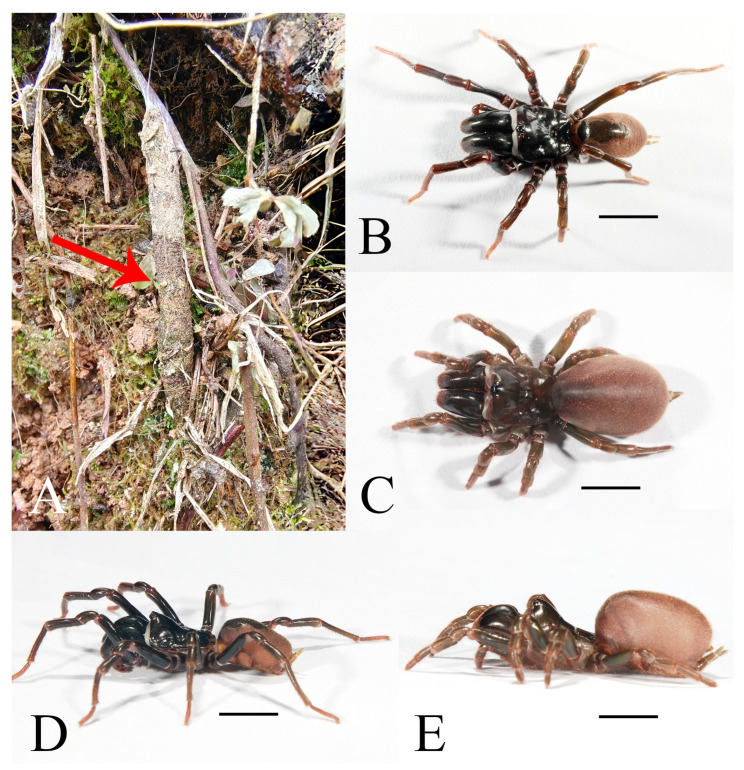
Microhabitat and general somatic morphology of *Atypus siyiensis* sp. nov. (**A**) purse-web (see red arrow point); (**B**,**D**) male (HNU1353); (**C**,**E**) female holotype (HNU1352). Scale bars: (**B**–**E**) 5 mm.

**Figure 6:**
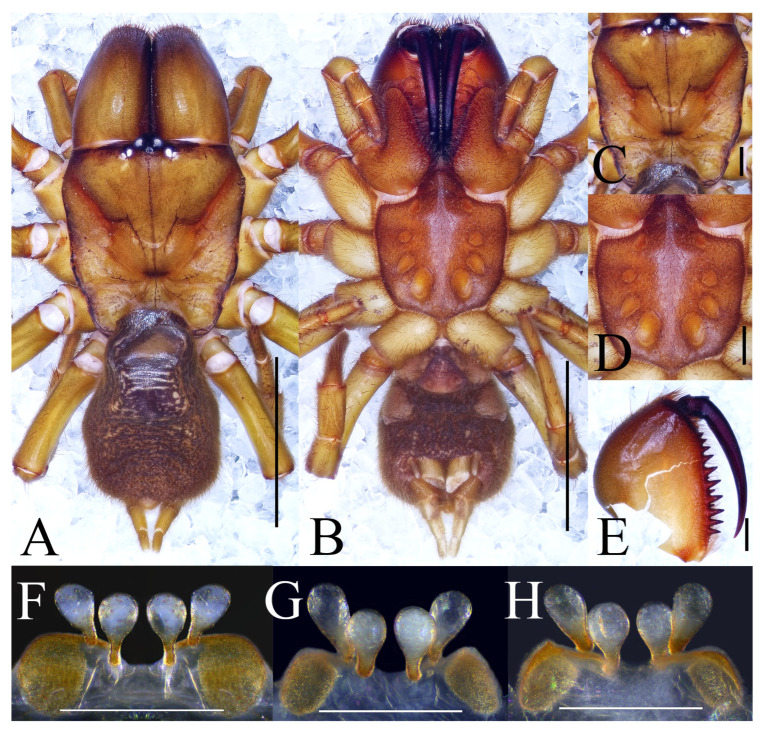
General somatic morphology and genital anatomy of *Atypus siyiensis* sp. nov. (**A**–**E**,**G**,**H**) female holotype (HNU1352); (**F**) (HNU1358). (**A**,**B**) habitus; (**A**) dorsal view; (**B**) ventral view; (**C**) carapace, dorsal view; (**D**) labium and sternum, ventral view; (**E**) left chelicera, inner-lateral view; (**F**,**G**) vulva, ventral view; (**H**) same, dorsal view. Scale bars: (**A**,**B**) 5 mm; (**C**–**E**) 1 mm; (**F**–**H**) 0.5 mm.

**Figure 7:**
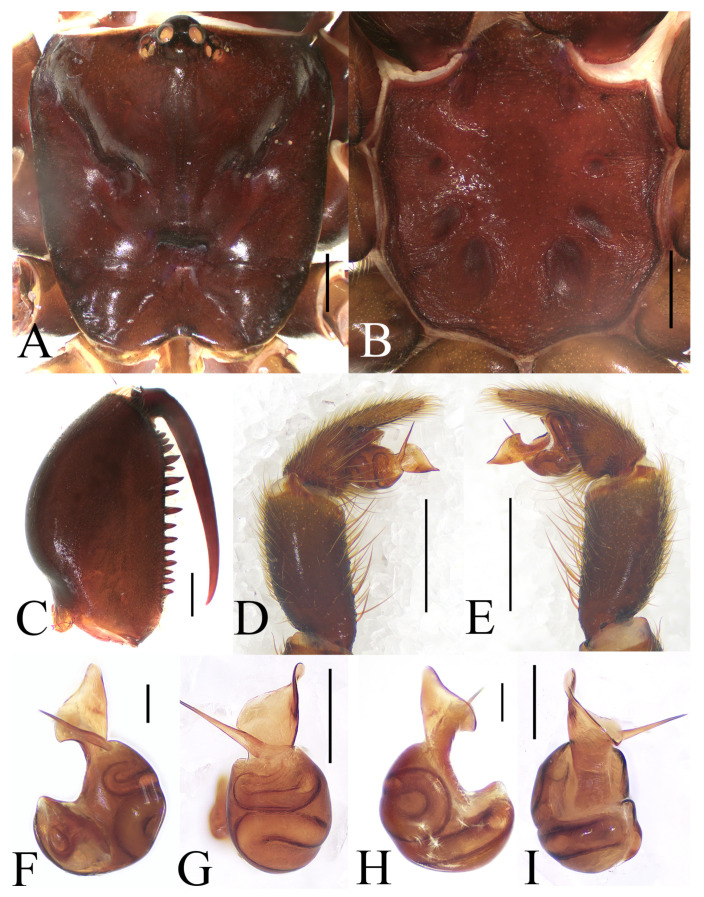
General somatic morphology and genital anatomy of *Atypus siyiensis* sp. nov. male (HNU1353). (**A**) carapace, dorsal view; (**B**) labium and sternum, ventral view; (**C**) left chelicera, inner-lateral view; (**D**) left palpal, prolateral view; (**E**) same, retrolateral view; (**F**) left palpal bulb, prolateral view; (**G**) same, ventral view; (**H**) same, retrolateral view; (**I**) same, dorsal view. Scale bars: (**A**–**E**) 1 mm; (**F**–**I**) 0.2 mm.

Figure 5, Figure 6 and Figure 7

Type material. Holotype female (HNU1352): CHINA: Sichuan Province, Chengdu City, Xindu District, Siyi Village, 30.80° N, 104.20° E, 53 m a.s.l., 18 October 2023, YC Wu leg.

Paratypes. Four females (HNU1354—HNU1357) and one male (HNU1353), same data as for holotype. One female (HNU1358), CHINA: Sichuan Province, Bazhong City, Nanjiang County, Guangwushan Town, Guangwushan Tourist Scenic Area, 32.68° N, 106.77° E, 1013 m a.s.l., 3 June 2022, AL He, JX Liu, ZG Huang, Y. Liang, Y. Hui, YL Wen, Y. Liu leg.

Etymology. The specific name refers to the type locality.

Diagnosis: The female of the new species resembles that of *A. suiningensis* Zhang 1985 in the shape of the receptacula (cf. Figure 6F–H with Zhu et al. 2006: figures 90–92 [17]) but can be distinguished by the spherical median pair of receptacula, the presence of prominent basal stalks, the anterior and posterior median eyes being almost equal in size, and the posterior edge of the sternum is not embedded between the bases of the fourth pair of legs (Figure 6C,D,F–H) (vs. all four receptacula pyriform and nearly equal in size, without prominent basal stalks, the anterior median eyes significantly larger than the posterior median eyes, and the posterior edge of the sternum slightly embedded between the bases of the fourth pair of legs in *A. suiningensis*) (see figures 87, 89–92 in Zhu et al. 2006 [17]).

Male of the new species resembles those of *A. suiningensis* and *A. sinensis* Schenkel 1953 in the shape of their palpal bulb (cf. Figure 7D–I with Zhu et al. 2006: figures 95–98 [17] and Zhu et al. 2006: figures 83–86 [17]). It can be distinguished from *A. suiningensis* by the conductor not wrapping around the embolus and the presence of an angle between conductor and embolus (cf. Figure 7D–I with Zhu et al. 2006: figures 95–98 [17]). It differs from *A. sinensis* by the latter having a thicker embolus, the first pair of sigilla positioned significantly closer to the margin of the sternum and the third pair of sigilla relatively bigger (cf. Figure 7B,D–I with Zhu et al. 2006: figures 82–86 [17]).

Description. Female (holotype). TL 13.94. CL 5.05, CW 5.27, AL 5.51, AW 3.93, FPC 1.68. Carapace yellow brown (Figure 6C). Fovea transverse, occupying about 1/6 of carapace width at the same horizontal level, R1 0.33×, Radial grooves distinct (Figure 6C). Eye diameter and inter-distances: AME 0.23, ALE 0.27, PME 0.20, PLE 0.21; AME–AME 0.34, AME–ALE 0.26, PME–PME 1.01, PME–PLE 0.04. MOA 0.50 long, front width 0.82, back width 1.27. Labium wider than long (Figure 6D). Sternum reddish brown, 4.17 long, 3.75 wide, moderately rough, and clothed with fine black hairs. Sigilla deeply imprinted, DFS 1.18, WFS 0.59, with the second pair rounded, the third pair teardrop-shaped, and the posterior pair oval, larger than other pairs, separated by nearly their transverse diameter; R2 2× (Figure 6D). Chelicerae yellow brown, with 16 teeth on promargin in a single row, the basal three fairly small (Figure 6E).

Abdomen oval, brownish, with indistinct oval dorsal scutum on anterior half (Figure 6A,B). Six spinnerets: ALS 0.68 long, PMS 1.02 long, four-segmented PLS with lengths as follows: basal 0.71, median 0.61, subapical 0.65, apical 0.57, total 2.54.

Legs stronger than those of the male, yellow brown (Figure 5C–E). Spines present on all metatarsi; metatarsus IV with 12 ventral spines. Leg formula: 4123. Leg lengths are provided in Table 3.

Vulva (Figure 6F–H): pore patches oval; receptacula attached to anterior edge of atrium; the anterior margins of lateral and median receptacula on two different horizontal lines; median receptacula with obvious basal stalks, and more rounded than lateral ones.

Male (holotype). CL 5.42, CW 5.05, FPC 1.75. Carapace black brown (Figure 7A). Fovea transverse, occupying about 2/11 of carapace width at the same horizontal level, R1 0.32x. Radial grooves distinct (Figure 7A). Eye diameter and inter-distances: AME 0.32, ALE 0.27, PME 0.20, PLE 0.18; AME–AME 0.26, AME–ALE 0.21, PME–PME 0.85, PME–PLE 0.04. MOA 0.55 long, front width 0.83, back width 1.08. Labium wider than long (Figure 7B). Sternum reddish brown, 3.81 long, 3.56 wide, relatively smooth, with sparsely distributed fine hairs. Sigilla deeply imprinted, DFS 1.08, WFS 0.49; second pair small and rounded; third pair olive-shaped; posterior pair oval, much bigger than other pairs, R2 2.2× (Figure 7B). Chelicerae black brown, with 19 teeth on promargin in a single row, the basal three fairly small (Figure 7C).

Abdomen dark brown, oval, with dorsal scutum gloss black, occupying about 3/4 of abdomen length (Figure 5B).

Legs slender in red grey (Figure 5B,D).

Male palp (Figure 7D–I): long conductor with a triangular folded part of its upper corner in retrolateral view; embolus long, thin spike.

Variation. Size range of females: carapace length 3.39–5.05, carapace width 4.65–5.27, total length 12.27–14.64, n = 6.

Habitat. Purse-webs were found attached to shrubs.

Distribution. Known only from the type locality, Sichuan, China.

Remarks. Due to the fact that the sole male specimen of *A. siyiensis* sp. nov. was consumed by a female during mating experiments, we are unable to provide as extensive morphological photos as we have for the male of *A. yaozu* sp. nov. Despite the incomplete male specimen, this does not detract from our assessment that the male and female specimens belong to the same species. Based on the K2P model, the pairwise distance between the female holotype and male specimens is 1.35%, and based on the *p*-distance model, it is 1.34%, both of which support the conclusion that these specimens of different sexes belong to the same species. Additionally, specimens of both sexes were collected from the same location at the same time.

*Atypus yanjingensis* sp. nov. (盐井地蛛)

urn:lsid:zoobank.org:act:997E0C76-1812-4B8E-9EE3-14E11C748677

**Figure 8:**
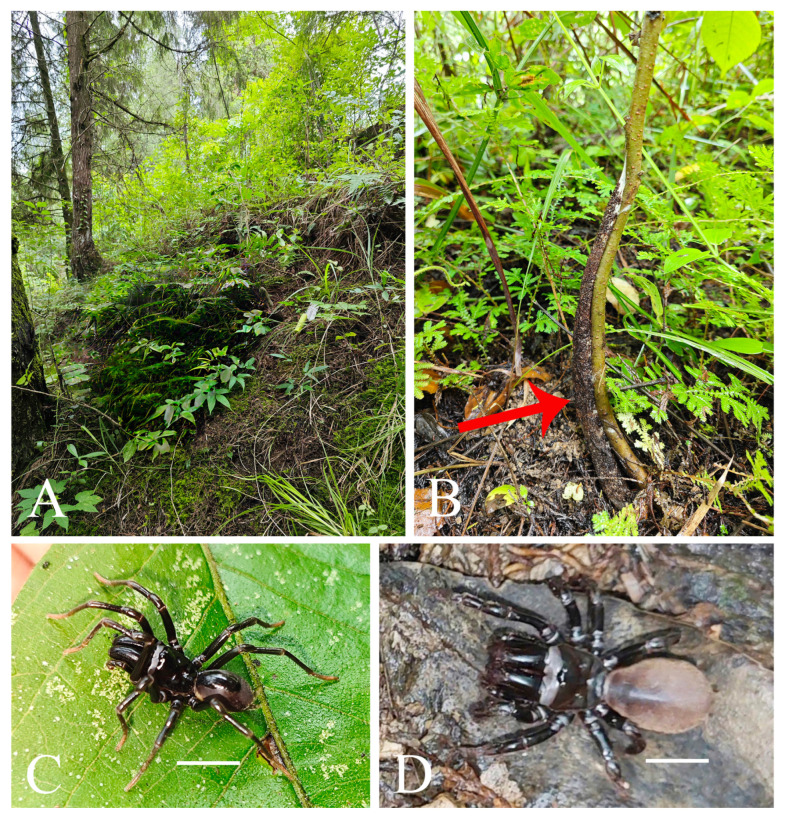
Microhabitat and general somatic morphology of *Atypus yanjingensis* sp. nov. (**A**,**B**) microhabitat; (**B**) purse-web (see red arrow point); (**C**) male (HNU1362); (**D**) female holotype (HNU1361). Scale bars: (**C**,**D**) 5 mm.

**Figure 9:**
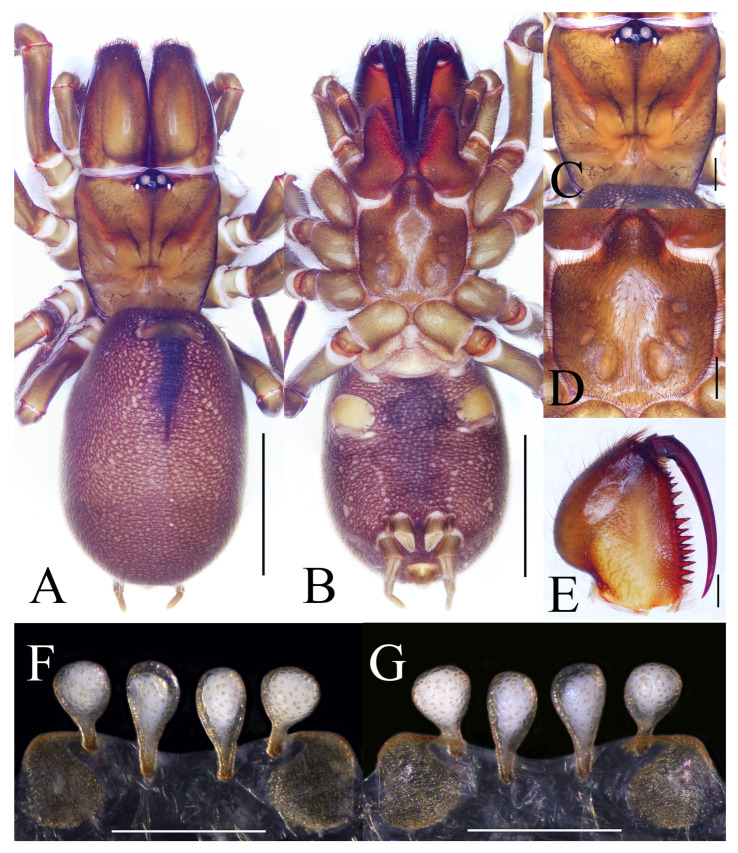
General somatic morphology and genital anatomy of *Atypus yanjingensis* sp. nov. female holotype (HNU1361). (**A**,**B**) habitus; (**A**) dorsal view; (**B**) ventral view; (**C**) carapace, dorsal view; (**D**) labium and sternum, ventral view; (**E**) left chelicera, inner-lateral view; (**F**) vulva, ventral view; (**G**) same, dorsal view. Scale bars: (**A**,**B**) 5 mm; (**C**–**E**) 1 mm; (**F**,**G**) 0.5 mm.

**Figure 10:**
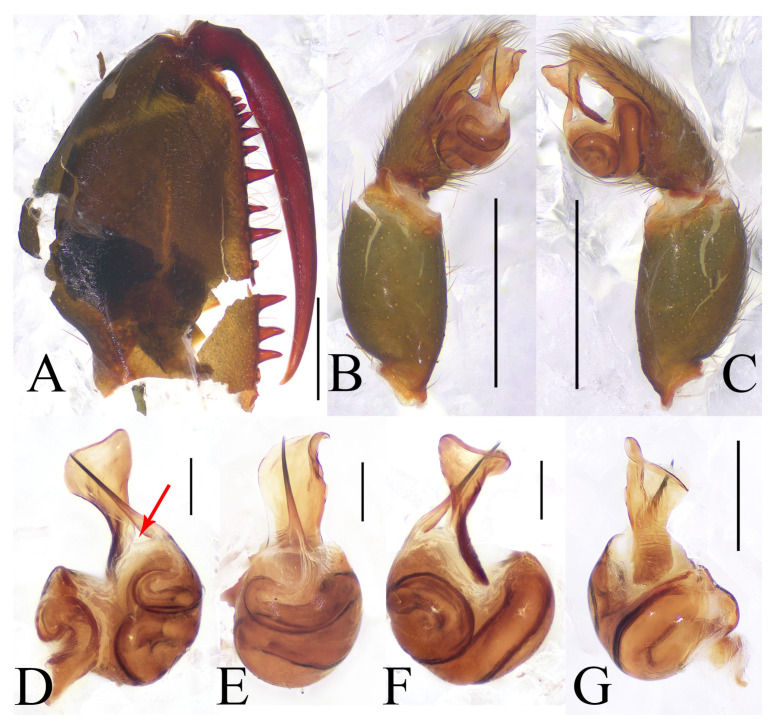
Chelicera and genital anatomy of *Atypus yanjingensis* sp. nov. male (HNU1362). (**A**) left chelicera, inner-lateral view; (**B**) left palpal, prolateral view; (**C**) same, retrolateral view; (**D**) left palpal bulb, prolateral view (red arrow indicates the triangular space between bases of embolus and conductor in lateral views); (**E**) same, ventral view; (**F**) same, retrolateral view; (**G**) same, dorsal view. Scale bars: (**A**–**C**) 1 mm; (**D**–**G**) 0.2 mm.

Figure 8, Figure 9 and Figure 10

Type material. Holotype female (HNU1361): CHINA: Hunan Province, Xiangxi Tujia and Miao Autonomous Prefecture, Yongshun County, Yanjing Village, 28.94° N, 109.73° E, 289 m a.s.l., 23 June 2024, YC Wu, Y. Liu, SX Zhu leg.

Paratypes. Five females (HNU1363—HNU1367) and one male (HNU1362), same data as for holotype.

Etymology. The specific name refers to the type locality.

Diagnosis: The female of the new species resembles those of *A. jianfengensis* and *A. pedicellatus* Zhu et al. 2006 in the shape of the receptacula (cf. Figure 9F,G with Li et al. 2018: figure 4D–E [18] and Zhu et al. 2006: figures 59–62 [17]) but can be distinguished by the shorter basal stalks of the median pair of receptacula and the first pair of sigilla being especially close to the margin of the sternum (Figure 9D,F,G) (vs. the basal stalks of the median pair of receptacula being noticeably longer and the first pair of sigilla not as close to the margin of the sternum in both *A. jianfengensis* and *A. pedicellatus*) (see figure 4B,D–E in Li et al. 2018 [18] and figures 58–62 in Zhu et al. 2006 [17]).

Male of the new species closely resembles that of *A. pedicellatus* in the shape of their palpal organ (cf. Figure 10B–G with Zhu et al. 2006: figures 65–67 [17]) but can be distinguished by the particularly low upper distal corner of the conductor and the strongly curved distal margin of the conductor (cf. Figure 10B–G with Zhu et al. 2006: figures 65–67 [17]).

Description. Female (holotype). TL 19.26. CL 5.02, CW 5.09, AL 9.60, AW 6.68, FPC 1.78. Carapace yellow brown (Figure 9C). Fovea transverse, occupying about 1/5 of carapace width at the same horizontal level, R1 0.35x. Radial grooves distinct (Figure 9C). Eye diameter and inter-distances: AME 0.31, ALE 0.22, PME 0.19, PLE 0.24; AME–AME 0.30, AME–ALE 0.18, PME–PME 1.06, PME–PLE 0.04. MOA 0.52 long, front width 0.92, back width 1.57. Labium wider than long (Figure 9D). Sternum orange brown, 4.65 long, 3.98 wide, relatively smooth, covered with fine black hairs. Sigilla deeply impressed, DFS 1.26, WFS 0.47; first pair positioned close to the margin of the sternum; second pair rounded; third pair elongated oval; posterior pair oval, larger than other pairs, separated by nearly their transverse diameter, R2 2.7× (Figure 9D). Chelicerae yellow brown, with 17 teeth on promargin in a single row, the basal one fairly small, with the first and seventh distal ones being relatively small (Figure 9E).

Abdomen oval, brownish, with distinct oval cardiac mark on anterior half (Figure 9A). Six spinnerets: ALS 0.56 long, PMS 1.20 long, four-segmented PLS with lengths as follows: basal 1.08, median 0.93, subapical 0.72, apical 0.79, total 3.52.

Legs stronger than those of the male, yellow brown (Figure 8D). Spines present on all metatarsi; metatarsus IV with 12 dorsal spines. Leg formula: 4123. Leg lengths are provided in Table 4.

Vulva (Figure 9F,G): pore patches large, rounded; lateral receptacula more rounded than median ones, both with distinct basal stalks; basal stalks of median receptacula longer than those of lateral ones, and all receptacula with their anterior margins almost on the same horizontal line.

Male (paratype). Carapace and chelicerae black brown (Figure 8C). Abdomen dark brown, oval, with dorsal scutum gloss black, occupying nearly 4/5 of abdomen length (Figure 8C).

Male palp (Figure 10B–G): upper distal corner of the conductor particularly low, distal margin of the conductor strongly curved; embolus long, thin spike with a small triangular space between embolus and conductor in lateral views.

Variation. Size range of females: carapace length 3.98–5.45, carapace width 4.33–5.09, total length 13.47–19.26, n = 6.

Habitat. Purse-webs were found attached to shrubs.

Distribution. Known only from the type locality, Yanjing Village (Yongshun County, Hunan Province, China).

Remarks. Male of this new species had the same situation as that of *A. siyiensis* sp. nov., consumed by a female. The pairwise distance between the female holotype of *A. yanjingensis* sp. nov. and the male is 0.65% based on the K2P model and 0.64% based on the *p*-distance model, supporting the conclusion that the male and female specimens belong to the same species. The specimens of two sexes were collected from the same location at the same time.

### 3.2. Genetic Distance

The average intraspecific genetic distance for the three new species described in this study is provided in Table 5. The results of the interspecific genetic distance analyses between samples listed in Dataset I are presented in Tables S2 and S3, based on *p*-distance and K2P models, respectively. In our results, the interspecific genetic distances based on the *p*-distance model ranged from 2.5% to 16.9%, while those based on the K2P model ranged from 2.5% to 19.4%. Different samples of the same species were grouped to estimate evolutionary divergence. Estimates of evolutionary divergence over sequence pairs between groups are provided in Table 6 and Table 7, based on *p*-distance and K2P models, respectively.

### 3.3. Natural History

#### 3.3.1. Web-Weaving

**Figure 11:**
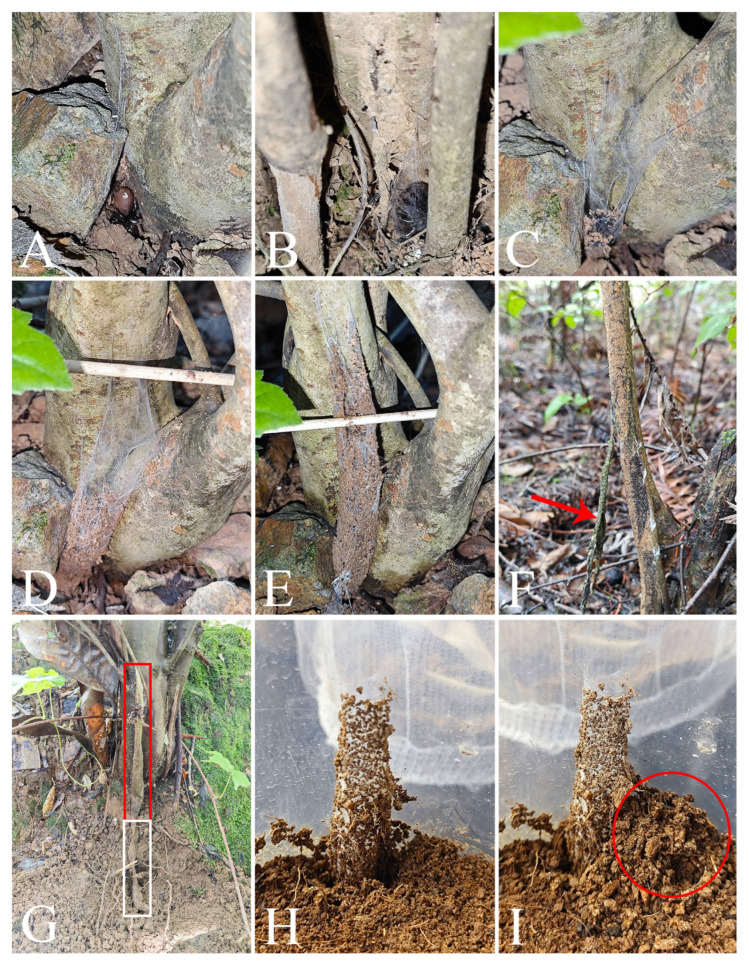
Web-weaving behavior of *Atypus* spiders. (**A**–**E**) weaving process; (**F**) new web and discarded old web (red arrow indicates the old web); (**G**) a complete tube (red box indicates the above-ground section, white box indicates the underground section); (**H**,**I**) soil pushed next to the above-ground section of the tube as it extends downward (red circle in (**I**) indicates the soil pushed out).

*Atypus* spiders live in underground burrows lined with silk and their silken tubes extend above ground (Figure 2B and Figure 11G). These tubes are typically anchored to nearby plant stems, walls or other available supports. The purse-web often begins at or just below the ground surface, where the spider excavates a small burrow, and weaves a longitudinally layer of silk around the surface of upright supports (Figure 11A,B). The spider reinforces the structure by repeatedly weaving silk vertically, section by section, extending upward along the tree (or other support) to form a distinct, tough silken tube (Figure 11B–E). This structure acts as a protective barrier, shielding the spider from harsh weather and predators while also serving as a trap for passing insects. According to our observation, an adult spider can complete a well-formed tube in just one day.

The above-ground section of the tube is typically camouflaged with small bits of moss, grass, or soil from the immediate surroundings (Figure 11E). We generally believed that these soil particles, mosses, etc. are caused by passing insects or other factors over time. However, in reality, they are attached by spiders during the process of weaving webs, at least according to our laboratory observations (excluding other factors). At the same time, we also believe that it is possible for the influence of insects or other factors to further enhance camouflage in the wild.

In our field collecting and laboratory rearing, we have observed that all purse-webs of *Atypus* spiders are consistently vertical to the ground against support (Figure 2B and Figure 11G,H), without exception. However, a contrasting observation was also reported in the literature, where the purse-webs were found lying flat on the ground (see figure 4D–F in Řezáč et al. 2022 [24]), although this situation is rarely documented. Gertsch and Platnick [5] previously suggested that purse-web orientation could differentiate *Atypus* (horizontal webs) from *Sphodros* (found only in North America and constructing vertical webs). Nonetheless, Schwendinger [25] later demonstrated that both genera have the ability to construct webs in either orientation. More recently, Řezáč et al. [24] proposed that this orientation is influenced by various environmental factors, including the availability of supports, camouflage needs, and prey accessibility.

Small piles of soil are occasionally found next to the above-ground section of the tube, indicating that the tube is likely being extended or deepened (Figure 11H,I). The spider is usually positioned at the base of the tube, with its head pointing upwards. These burrows are commonly found on sunny slopes, where the above-ground section of the web is exposed to sunlight. The entrance is located at the end of the above-ground portion, with the side attached to support being higher and the opposite side (free side) lower, potentially aiding in preventing water from flooding the burrow during rain.

The length of the above-ground section is more variable compared to the underground part. Based on the limited data, we think that the length of the web is not correlated with spider body size, and we guess that it may be correlated to the duration of the spider’s residence within it. The longer the spider resides in the web, the more it reinforces and extends the structure, resulting in a progressively longer web. Additionally, the length of the web may also be influenced by the abundance of prey in the environment. When prey near the base of the tree is insufficient to meet the spider’s dietary needs, the web may continue to extend upward to increase hunting opportunities. Previously, Řezáč et al. [24] also reported that web length (both subterranean and above-ground) and the ratio of subterranean to above-ground segments were not correlated with spider body size. The longest recorded purse-web to date features an above-ground section measuring 64.5 cm and an underground section measuring 14 cm, giving it a total length of 78.5 cm and a diameter of 21 mm [26].

Although there is no evidence to support a correlation between web length and spider size, web diameter does indeed show to be related to body size. As a spider ages, its purse-web increases in both size and toughness, with the tube diameter expanding proportionally to the spider’s growth. Rather than expanding the diameter of the existing web to accommodate its growing bodies, the spider constructs a new web. This process begins with the spider making a longitudinal cut in the above-ground portion of the old web. Then, it emerges and starts building a new web either against or next to the old one. The spider continues to reside in the old web until the new one is completed. Once the new web is finished, the spider abandons the old one (Figure 11F).

#### 3.3.2. Predatory Strategy

*Atypus* species weave numerous irregular silk lines around the aerial tube. When insects or other small animals approach and touch these lines, the vibrations they produce alert the spider inside the tube. The spider then positions itself beneath the prey and keeps a longitudinal distance from the prey outside, assessing whether to attack. If the approaching prey is small and moves slowly, the spider usually initiates a predatory response. However, if the prey is significantly larger or moves rapidly, the spider generally refrains from attacking. The spider’s decision to hunt or not is also influenced by its condition; for instance, well-fed spiders are less likely to attack, and female spiders that have recently mated may mistake approaching males as prey.

**Figure 12:**
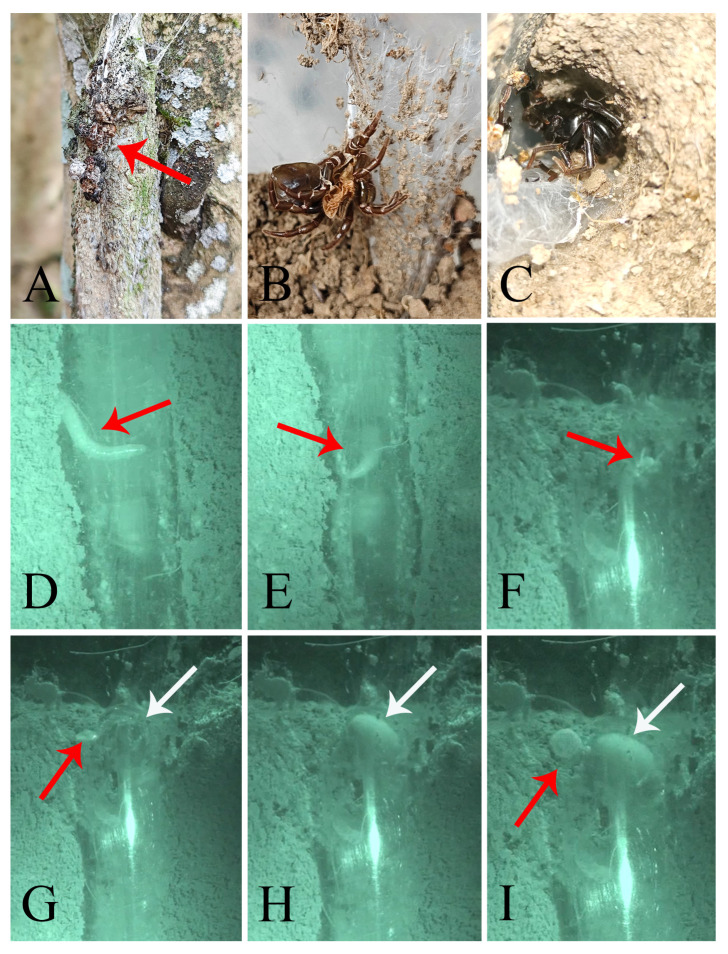
Feeding and defecation processes of *Atypus* spiders. (**A**) remains of prey discarded outside the web (see red arrow point); (**B**) exuvium; (**C**) male spider expelled after being consumed; (**D**) prey crawling over the aerial tube (see red arrow point); (**E**) feeding spider (see red arrow point); (**F**) remains of prey carried to the web entrance using the chelicerae (red arrow); (**G**) remains pushed out of the web after tearing it with the front legs (red arrow indicates the expelled remains; white arrow indicates the spider); (**H**) spider positioning its abdomen toward the web opening, preparing to defecate (white arrow); (**I**) defecation (red arrow indicates expelled droppings; white arrow indicates the spider).

After carefully assessing the situation, if the spider decides to attack the insects or other small animals crawling over the aerial tube, it swiftly pierces through the silk web and impales the prey with its long, slender fangs (Figure 12D,E). Then, it enlarges the silk opening just enough to drag the prey inside. Once inside, it injects venom through its fangs to paralyze the prey, wraps it in silk, and repairs the torn section of the tube before consuming its meal. After feeding, the spider uses its cheliceral fangs to expel the remains (usually the exoskeleton) of the prey from the silk tube through a temporary opening in minority cases or the entrance at the top of the tube in majority cases (Figure 12F,G). Remnants of previous prey are usually found just outside the entrance in both field and laboratory settings (Figure 12A).

The remarkable and unique predatory technique of *Atypus* spiders was first demonstrated by Enock [27]: “The spider lies in ambush within or near the aerial portion of its tubular web. Upon sensing prey, it swiftly pierces the web with its long fangs to capture it. The prey is then secured with silk threads. In most cases, the spider begins repairing the torn web shortly after immobilizing the prey, pulling the torn edges together and spinning silk across the rent. However, in some instances, the spider may prioritize feeding and delay repairing the web for up to a day. After consuming the prey, the spider discards the remnants outside the tube, and prey fragments can often be observed attached to the web surface.” This predatory behavior has been corroborated many times since [5,15,16,27,28,29,30]; our observations have not only confirmed these findings but also provided additional details.

#### 3.3.3. Defecation Behavior

Atypids never defecate within their silk tube. Instead, they move cautiously toward the top entrance, progressing slowly and intermittently while frequently pausing to assess their surroundings for potential threats. Upon reaching the entrance, they deftly use their forefoot to gently open it. Then, with a swift motion, they turn their bodies and position the end of their abdomen towards the opening. Slowly extending the end of their abdomen outward, they swiftly eject white liquid from their anus (Figure 12H,I). The ejected droppings solidify within minutes after being expelled.

#### 3.3.4. Defensive Behavior

**Figure 13:**
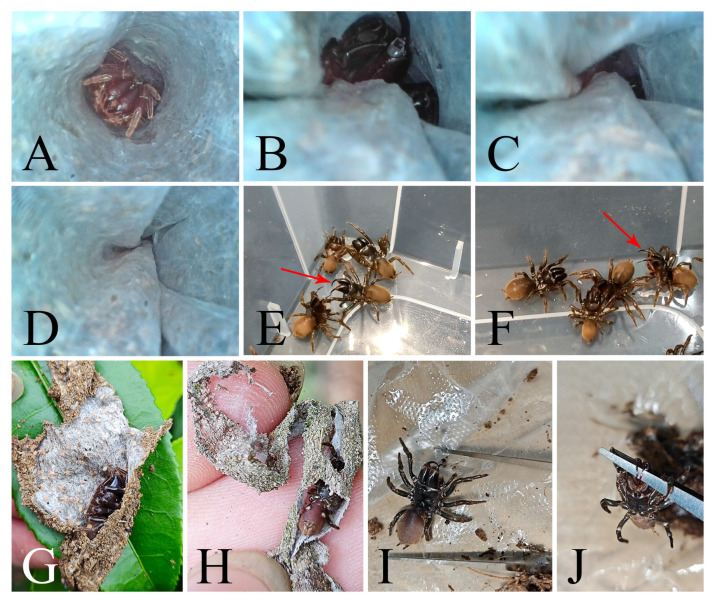
Defensive behavior of *Atypus* spiders. (**A**) *Atypus* spider inside its web; (**B**–**D**) spider responds to an attack from within the web by using its chelicerae to quickly grasp and twist the web, sealing off the lower portion for protection; (**E**,**F**) individuals raising their chelicerae high to intimidate or attack others when multiple spiders are placed together (red arrows indicate the raised chelicerae); (**G**) spider retreats to the remaining portion of its web after the web is torn; (**H**) even when the web is completely shredded, the spider remains within and attempts to repair it; (**I**) spider raises its chelicerae high when provoked with tweezers; (**J**) spider grasping the tweezers with its chelicerae.

*Atypus* spiders are typically sedentary, residing within their tube webs to await and ambush approaching prey (Figure 13A). Their unique silk tubes not only serve as dwellings but also facilitate an extraordinary defensive mechanism. In the event of an attack originating from above within the web, these spiders swiftly descend into the subterranean portion of their web and utilize their chelicerae to grasp and twist a section of the silk, effectively sealing off the lower region of the tube from the threat (Figure 13B). This strategy isolates them from danger and evades potential attacks.

Attempts to displace *Atypus* spiders from their webs often are not easy. They strive to retreat deeper into their webs, even if the structure has been torn into fragmented sections, and they still diligently attempt to repair it (Figure 13G,H).

When forcibly removed from their webs and provoked with a stick or tweezers, the spiders adopt a defensive stance, raising their chelicerae high in a threatening posture (Figure 13I). Direct contact with tweezers elicits an immediate response: the spiders latch onto the tool with their fangs (Figure 13J), a behavior also observed during their prey-hunting activities. In multi-spider encounters, they utilize their fangs to intimidate or defend against one another (Figure 13E,F), typically in response to the proximity of other spiders rather than as an aggressive act.

#### 3.3.5. Life-History

**Figure 14:**
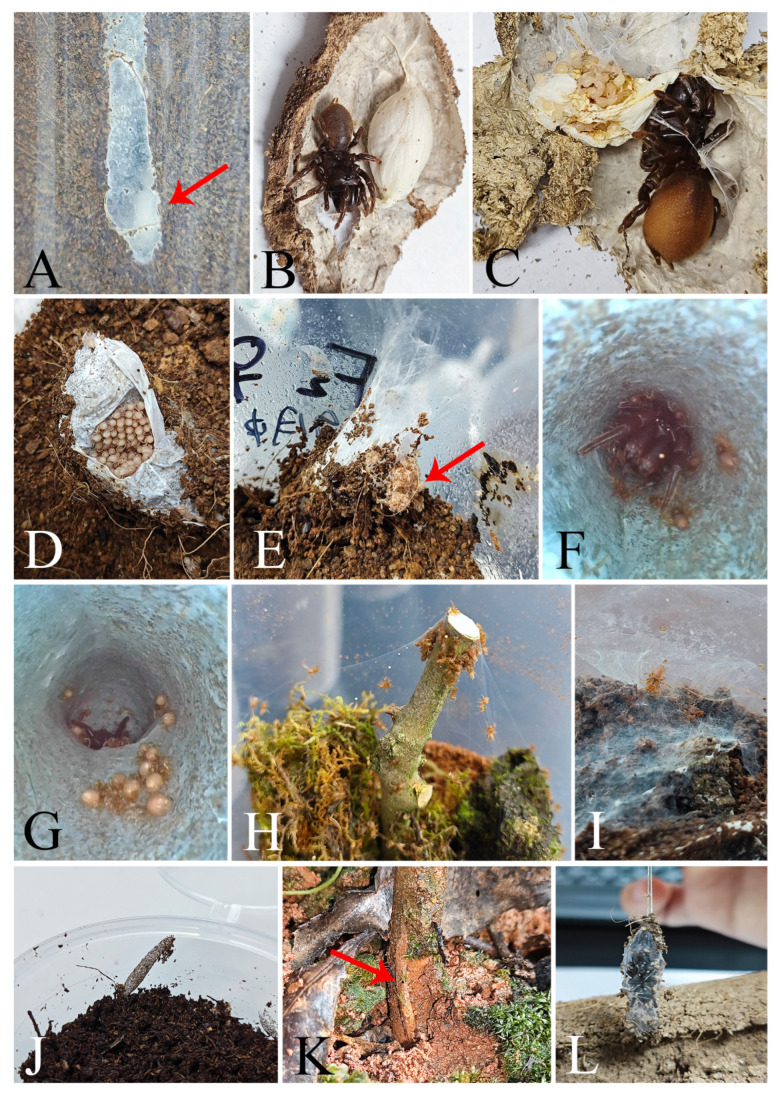
Developmental stages and dispersal behavior of *Atypus* spiders. (**A**) spider in the tube (red arrow indicates the egg sac); (**B**) female spider with her egg sac; (**C**) female spider with newly hatched first instar juveniles; (**D**) first instar juveniles hiding in the web; (**E**) the exuviae pushed out of the tube (red arrow indicates the exuviae); (**F**,**G**) juveniles living inside the maternal web; (**H**) early instar juveniles dispersing by “gossamer”; (**I**) relatively complete web woven by a third instar *Atypus* spider; (**J**,**K**) tubular webs woven by post-third instar juveniles: (**J**) photographed under laboratory conditions; (**K**) photographed under natural conditions; (**L**) thin tubular web woven by a male *Atypus* spider.

In mid-May in southern China, male specimens undergo their final molt, marking the onset of sexual maturity. During this period, mature males can be collected in the field, along with some freshly molted, incompletely developed males are also found. In natural settings, males reach maturity between mid-May and early June and subsequently leave their webs to seek females for mating. By mid- to late-June, a portion of the females (although no specific data can be provided) have already mated, and mature males can rarely or hardly be collected in the field. Additionally, during this period, some tubes were found empty, further indicating the males’ departure. In laboratory conditions, collected females, regardless of their maturity, burrow and weave new purse-webs. However, mature males exhibit different behavior: they no longer burrow or weave complete purse-webs, although they retain the ability to produce silk. Interestingly, if a pit is dug in advance under laboratory conditions, mature males will spin silk and weave very thin, tubular webs within the pit (Figure 14L). It is noteworthy that these webs do not extend upwards beyond the excavation plane. These behaviors of mature male spiders are believed to be energy-saving strategies for the upcoming mating season. Furthermore, our observations indicate that all male spiders cease feeding after reaching maturity.

After mating, the female may kill the male or allow him to coexist peacefully in her tube. Our observations indicate that this coexistence can last up to 22 days. However, longer durations have been reported, with Gertsch and Platnick [5] noting periods of several months and Hiebsch and Krause [29] recording coexistence of up to nine months inside the tubes of their mates in *Atypus affinis*. Following this period, the adult female lays her eggs. The number of eggs typically ranges between 150 and 200 in our observations, with a record of up to 441 eggs noted by Schwendinger [2]. These eggs are carefully placed in an olive-shaped sac, which is usually suspended just inside the underground section of the tube. The sac is securely attached to the nest wall at both ends by thin stalks, ensuring it remains firmly fixed to one side of the tube (see Figure 14A,B). Additionally, we have observed that *Atypus* spiders continue to molt even after laying their eggs.

**Figure 15:**
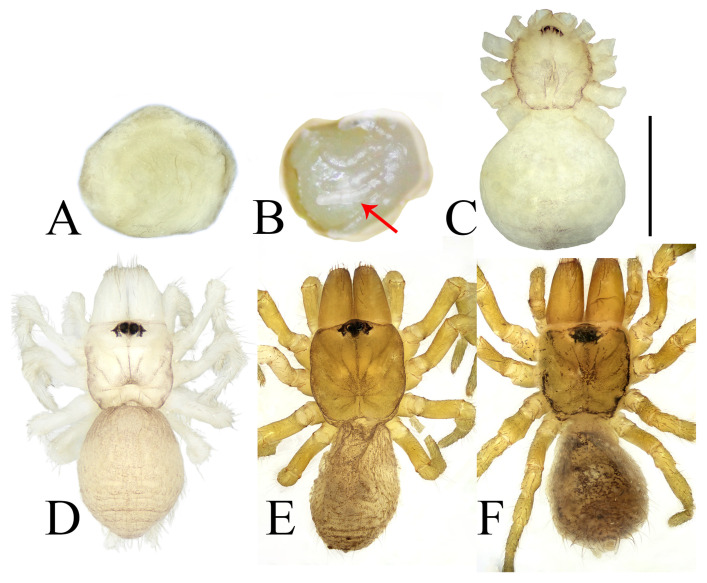
Changes in appearance from egg to third instar juvenile. (**A**) egg; (**B**) egg in the process of hatching (red arrow indicates legs extending from the egg); (**C**) first instar juvenile, dorsal view; (**D**) second instar juvenile, dorsal view; (**E**) third instar juvenile, dorsal view; (**F**) third instar juvenile after one month, dorsal view. Scale bars: (**A**–**F**) 0.5 mm.

From August, the young hatch but remain in the tube, guarded by their mother (Figure 14C). They overwinter in the maternal tube (Figure 14F,G) and disperse to start their own burrows in the following spring. Approximately 15–16 days after the eggs are laid, the spiderlings hatch as first instar juveniles. At this early stage of development, the spiderlings’ cephalothorax and legs emerge from the eggs, while the original eggs form the abdomens. Notably, the abdomen is significantly larger than the cephalothorax at this point (Figure 15B,C). These first instar juveniles are highly sensitive to light, have limited mobility, and are characterized by constricted chelicerae. Upon being removed from the egg sac, they tend to cluster together and retreat into the tube for shelter (Figure 14D). If exposed without shelter, they burrow into the soil and become less active, which could severely reduce their survival rate. Our observations also reveal that spiderlings are capable of hatching from the web even in the absence of their mother, indicating that her presence is not essential for hatching. Eggs taken from the sac can successfully hatch under artificial conditions, provided that the web’s cover is retained to maintain a dark environment and sufficient moisture. It is possible that other substitute coverings could yield similar results, although this was not tested in our study. Remarkably, even in the absence of their mother, the juveniles are capable of completing subsequent molts without feeding until they reach the third instar, presumably due to the nutrients stored in their disproportionately large abdomens.

About two weeks after hatching of eggs, the first true molt occurs, developing to the stage of second instar juveniles. At this stage, the mother spider pushes the shed skin (exuviae) and the empty egg sac out of the tube (Figure 14E). Second instar juveniles display slight pigmentation that gradually darkens over time (Figure 15D), and their mobility also improves. They started producing silk, though they had not yet formed extensive webs. Both first and second instar juveniles do not require food, likely due to their underdeveloped structures and limited predatory capabilities.

**Figure 16:**
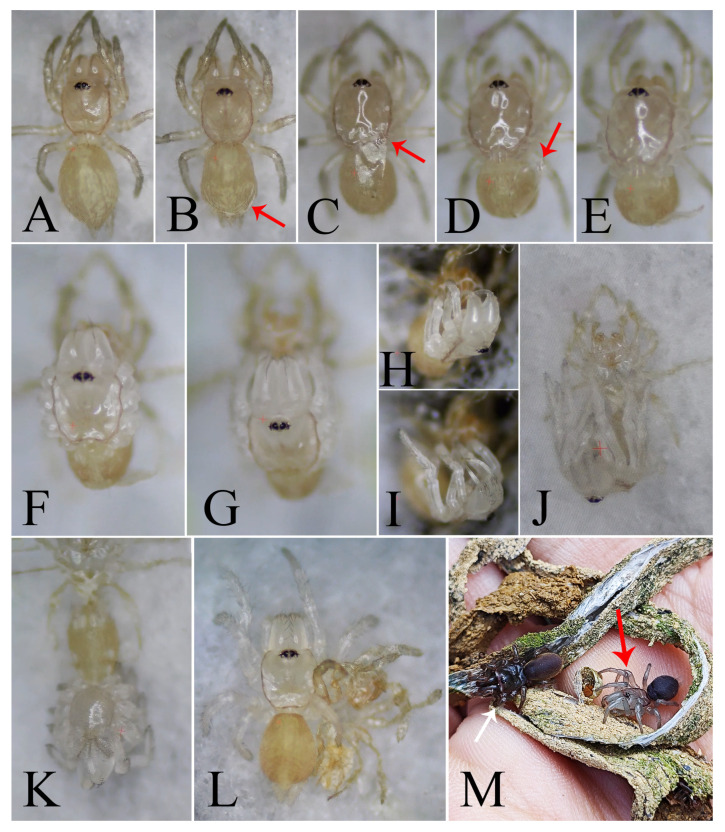
Molting process of *Atypus* spiders. (**A**–**L**) the transition of a second instar juvenile molting into a third instar: (**A**) spider before molting; (**B**) separation of the old exoskeleton from the spider (red arrow indicates wrinkles caused by the detachment of the old exoskeleton); (**C**) old carapace lifting visibly from the cephalothorax (red arrow indicates the raised carapace); (**D**) old exoskeleton splitting open along the carapace (red arrow indicates the detached carapace); (**E**–**J**) legs and chelicerae gradually emerging from the old exoskeleton; (**K**) spider lying on its back after completing molting; (**L**) third instar juvenile regaining an upright position a few minutes after molting, alongside the shed exoskeleton; (**M**) temporary transparency of the cephalothorax and legs commonly observed in *Atypus* spiders post-molting (red arrow indicates a freshly molted spider; white arrow indicates a spider with pigmentation after a period following molting).

Approximately three weeks after the previous molt, the spider undergoes another molt within the maternal tube web. During this process, the spider remains stationary as its old exoskeleton gradually separates. The old carapace visibly lifts from the cephalothorax, while distinct wrinkles appear on the abdomen (Figure 16A,B). About 20 min into the process, the old exoskeleton suddenly splits open along the margins of both the carapace and abdomen, with the old dorsal exoskeleton of the carapace connected to that of the abdomen by the pedicel (Figure 16C). Following this, the spider’s body begins to bend towards its ventral side and then bends more strongly, aiding in the emergence of the body from the old exoskeleton. Subsequently, legs and chelicerae gradually emerge (Figure 16E–I). Simultaneously, the abdominal exoskeleton loosens and peels away. Once the tarsi, chelicera fangs, and spinnerets are freed from the old exoskeleton, the molting process concludes, lasting approximately 50 min in total molting. After molting, the spider typically lies on its back but regains a normal position (with its back up) within a few minutes, becoming mobile (Figure 16K,L). Just after molting, the cephalothorax, chelicerae and legs appear transparent, but they gradually darken over time due to pigmentation—a phenomenon consistently observed in *Atypus* spiders before they reach maturity (Figure 16M).

By the third instar (after undergoing two molts), the juveniles have developed noticeably darker pigmentation (Figure 15E) and can construct more robust webs (Figure 14I), although they are still thinner than those of adult spiders. At this stage, they begin to feed. Changes in appearance from egg to third instar juvenile are provided in Figure 15. Juveniles after the third instar, when removed from the maternal web and reared individually, are capable of weaving their own tubular webs (Figure 14J). However, under natural conditions, juveniles at this stage typically remain within the maternal web until the following spring. This behavior may be attributed to the cold winter season, which is not conducive to juvenile dispersal.

Early instar juveniles disperse by “gossamer” (Figure 14H), a phenomenon described by Enock [27] as the “ballooning of juveniles”. The juveniles leave the maternal tube in single file, leaving a silk dragline as they follow one another up into bushes or trees, where they begin constructing small purse-webs of their own (Figure 14K).

The number of molts that an *Atypus* spider undergoes before reaching maturity remains uncertain. However, it has been established that male spiders do not feed after maturity and have a relatively short lifespan, while mature female spiders continue to molt and have a longer lifespan. Under laboratory conditions, we have recorded instances where a male spider lived for approximately two months (from 19 January 2024 to 24 March 2024) after its last molt. In another case, a female spider was raised for about seven years (after being collected from the field). Based on this, the literature mentioned above that female spiders can live for up to 8–10 years [2] is reliable.

Remarks: The timing of mating, egg-laying, juvenile hatching, molting and maturation in *Atypus* spiders appears to vary with local climate and species differences, as evidenced by variations observed among *Atypus* specimens collected from different regions. For instance, some males have been observed to mature as early as February.

## 4. Discussion

### 4.1. Morphology

After examining numerous *Atypus* specimens and consulting the previous literature, we have confirmed the diagnostic value of sigilla, taking into account their shapes, sizes, and relative positions. In this study, we introduce a new diagnostic feature: the ratio (R2) of the distance between the fourth pair of sigilla (DFS) to the width of the fourth sigilla (WFS) (as shown in Figure 1C). Additionally, we have identified two other effective data characteristics for taxonomic identification: the ratio (R1) of the distance between the fovea and the posterior end of the carapace (FPC) to the total length of the carapace (CL) (see Figure 1A), and the length-to-width ratio of the sternum (illustrated in Figure 1).

Compared to females, males of different *Atypus* species exhibit less pronounced interspecific variations. Consequently, female genitalia are generally more reliable than male palps for species identification within the genus *Atypus* [2,15,25]. However, identifying *Atypus* species based solely on morphological characteristics remains challenging. Yin [31] noted that intraspecific variation is common within the order Araneae, with primitive species displaying greater variation than modern ones. *Atypus*, as one of the primitive spider groups, has relatively simple morphological features characterized by high intraspecific variation and low interspecific differences. The primary diagnostic characteristic, genital morphology, is often inconsistent. For example, females of *Atypus heterothecus* Zhang, 1985 display variations in the shape of the receptacula and the length of the basal stalks among individuals [20,32]. Therefore, species identification within this group is particularly difficult, especially for those species lacking high-quality illustrations. In this study, we report and describe three new species, utilizing both morphological characteristics and molecular data for their identification.

The integration of molecular data and morphological features is undoubtedly an effective approach for species identification. However, it is important to note that due to the challenges in morphologically identifying *Atypus* species, there may be mismatches between the barcodes retrieved from NCBI, particularly as some barcodes are not derived from type specimens and are submitted directly without accompanying morphological information.

### 4.2. Introduced Species

The dispersal ability of *Atypus* spiders is highly limited, with females typically residing permanently within their purse-webs [5], while males only venture out during the mating season to seek out females. Theoretically, under natural circumstances, the distribution range of each species should be quite restricted. However, *Atypus* spiders tend to attach their webs to the stems of plants like tea trees, fir trees, and Sichuan pepper trees. This behavior enables them to indirectly expand their range through human activities, such as the planting and transplantation of plant seedlings. Consequently, the known distribution range of *Atypus* species may not accurately reflect their natural distribution. For example, in countries or regions with advanced tea cultivation, there may be a certain number of introduced *Atypus* species accompanying the introduction of tea trees.

In our experience, there are cases that can only be explained by acknowledging certain species as introduced species (specific details will be provided in the forthcoming article). Recently, Řezáč et al. [24] proposed that the only species of *Atypus* reported in North America, *A. snetsingeri* Schwendinger, 1990, is actually an introduced species. Based on genetic data and habitat requirements, they argued that *A. snetsingeri* should be regarded as a junior synonym of *A. karschi*. They demonstrated that *A. snetsingeri* from Pennsylvania is genetically conspecific with *A. karschi*, native to East Asia and first described in Japan. They speculated that *A. snetsingeri* was likely introduced to Pennsylvania by humans, possibly via potted plants, and has since become naturalized, now locally common in a limited range encompassing both urban and forested areas.

**Figure 17:**
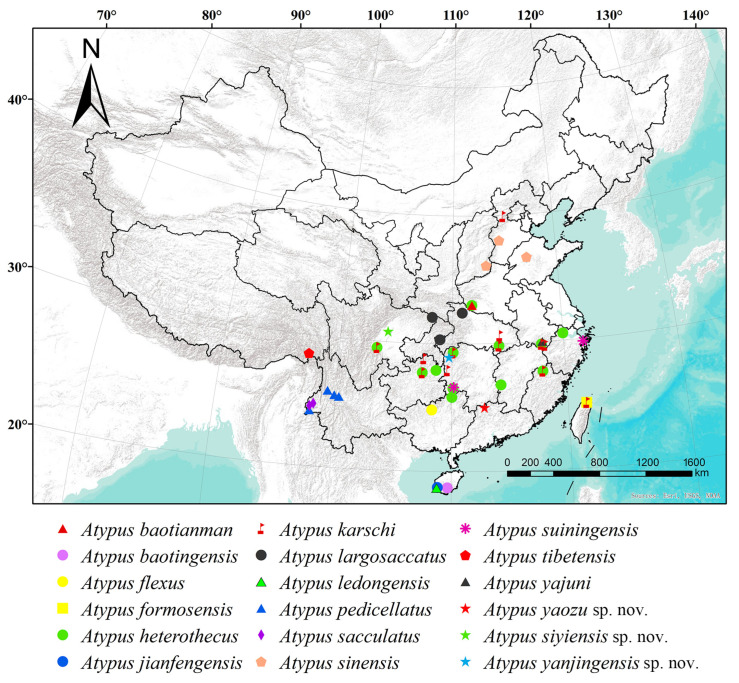
Distribution map of *Atypus* species in China, including the known species and the three new species described in this study.

Including the three new species reported in this study, a total of 18 *Atypus* species are now known to occur in China (WSC 2025). Most of these species are confined to southern China and have limited distribution ranges. Only *A. karschi* Dönitz, 1887 and *A. heterothecus* are considered to have wider distributions (Figure 17). *A. karschi*, in particular, has been recorded in several countries, including the USA (where it is considered introduced), China, Korea and Japan. However, the true extent of the distribution of these two species within China remains to be further verified. Additionally, the presence of *A. karschi* in China still requires confirmation.

### 4.3. Mating Behavior

Bertkau [10] observed that male *Atypus* spiders appear to be much rarer and proposed the possibility that a single might fertilize multiple females. Gerhardt [33] conducted a study on the sexual biology of the German species *Atypus muralis* Bertkau, 1890, describing the mating behavior: “After the wandering male finds the aerial web of the female, he drums on it with his legs and palpi, insuring that the waiting female is receptive, then releases a large drop of saliva onto the web to soften it. Once entry into the tube is accomplished, the male crawls beneath the female body and inserts his palps into the female’s genital groove alternately”.

In our laboratory observations, we noted varying behaviors of the mature male upon entering the female’s silk tube. Sometimes, he immediately escapes; sometimes, he stays for a few minutes and then flees in panic. In some instances, he remains inside for several hours before being killed by the female. The most intriguing observation, however, is when the male stays inside the silk tube with the female for up to 22 days. During this period, we often observed the female and the male exchanging positions within the tube. When the male is positioned nearer to the entrance than the female, he has a good opportunity to escape, yet he does not. Remarkably, after 22 days, we observed the exoskeleton of the male being pushed out of the silk tube. We speculate that in the final stages of the male spider’s life, the female spider consumes him as a source of nutrients to support her pregnancy. This behavior could potentially be interpreted as a form of altruistic self-sacrifice by the male, facilitating the successful reproduction of the next generation. However, this hypothesis remains to be tested through further research and experimentation.

## Figures and Tables

**Table 1:** Leg measurements of *Atypus yaozu* sp. nov., male.

	Femur	Patella	Tibia	Metatarsus	Tarsus	Total
I	3.83	1.62	2.31	3.01	1.87	12.64
II	3.27	1.45	1.83	2.51	1.65	10.71
III	2.81	1.28	1.53	1.92	1.56	9.1
IV	3.49	1.20	1.98	3.41	2.30	12.38

**Table 2:** Leg measurements of *Atypus yaozu* sp. nov., female.

	Femur	Patella	Tibia	Metatarsus	Tarsus	Total
I	3.89	2.18	1.85	2.15	1.28	11.35
II	3.49	2.20	1.61	1.99	1.23	10.52
III	3.19	1.97	1.23	1.83	1.10	9.32
IV	3.91	2.08	1.83	2.44	1.40	11.66

**Table 3:** Leg measurements of *Atypus siyiensis* sp. nov., female.

	Femur	Patella	Tibia	Metatarsus	Tarsus	Total
I	3.79	1.97	1.66	2.02	1.21	10.65
II	3.23	1.87	1.41	1.60	1.24	9.35
III	2.56	1.58	1.11	1.50	1.18	7.93
IV	3.38	1.90	1.78	2.32	1.33	10.71

**Table 4:** Leg measurements of *Atypus yanjingensis* sp. nov., female.

	Femur	Patella	Tibia	Metatarsus	Tarsus	Total
I	4.40	2.20	2.02	1.80	1.03	11.45
II	3.68	1.88	1.64	1.53	1.20	9.93
III	2.97	1.50	1.38	1.89	1.19	8.93
IV	3.79	2.04	1.85	2.49	1.55	11.72

**Table 5:** Estimates of average evolutionary divergence over sequence pairs within groups of the three new species in this study (based on both *p*-distance and K2P).

Species	Distance (Based on *p*-Distance)	Distance (Based on K2P)
*Atypus yanjingensis* sp. nov.	0.0037	0.0037
*Atypus yaozu* sp. nov.	0.0089	0.0090
*Atypus siyiensis* sp. nov.	0.0071	0.0071

**Table 6:** Estimates of evolutionary divergence over sequence pairs between groups of all available samples from China (based on *p*-distance).

	1	2	3	4	5	6	7	8	9	10
*A. baotianmanensis*		0.0129	0.0152	0.0130	0.0121	0.0126	0.0136	0.0123	0.0138	0.0131
*A. baotingensis*	0.1161		0.0094	0.0130	0.0134	0.0138	0.0140	0.0128	0.0134	0.0124
*A. ledongensis*	0.1466	0.0683		0.0144	0.0149	0.0153	0.0167	0.0153	0.0153	0.0135
*A. yanjingensis* sp. nov.	0.1232	0.1343	0.1401		0.0131	0.0133	0.0141	0.0125	0.0143	0.0133
*A. jianfengensis*	0.1146	0.1383	0.1528	0.1389		0.0114	0.0141	0.0123	0.0140	0.0136
*A. yaozu* sp. nov.	0.1231	0.1479	0.1683	0.1409	0.1048		0.0134	0.0122	0.0135	0.0138
*A. sacculatus*	0.1137	0.1285	0.1571	0.1243	0.1242	0.1154		0.0061	0.0145	0.0140
*A. yajuni*	0.1207	0.1324	0.1632	0.1241	0.1268	0.1195	0.0329		0.0133	0.0127
*A. heterothecus*	0.1316	0.1291	0.1571	0.1505	0.1424	0.1347	0.1385	0.1416		0.0122
*A. siyiensis* sp. nov.	0.1248	0.1269	0.1446	0.1312	0.1467	0.1477	0.1250	0.1307	0.1103	

**Table 7:** Estimates of evolutionary divergence over sequence pairs between groups of all available samples from China (based on K2P).

	1	2	3	4	5	6	7	8	9	10
*A. baotianmanensis*		0.0146	0.0194	0.0160	0.0149	0.0161	0.0159	0.0144	0.0168	0.0161
*A. baotingensis*	0.1276		0.0109	0.0162	0.0161	0.0175	0.0166	0.0155	0.0159	0.0153
*A. ledongensis*	0.1668	0.0725		0.0175	0.0186	0.0204	0.0212	0.0196	0.0198	0.0182
*A. yanjingensis* sp. nov.	0.1369	0.1495	0.1568		0.0168	0.0173	0.0171	0.0153	0.0182	0.0160
*A. jianfengensis*	0.1251	0.1536	0.1722	0.1557		0.0140	0.0163	0.0147	0.0168	0.0173
*A. yaozu* sp. nov.	0.1360	0.1660	0.1931	0.1578	0.1145		0.0157	0.0148	0.0165	0.0177
*A. sacculatus*	0.1238	0.1414	0.1778	0.1365	0.1366	0.1264		0.0065	0.0175	0.0166
*A. yajuni*	0.1328	0.1465	0.1863	0.1366	0.1405	0.1319	0.0339		0.0164	0.0157
*A. heterothecus*	0.1457	0.1424	0.1780	0.1697	0.1587	0.1497	0.1539	0.1581		0.0146
*A. siyiensis* sp. nov.	0.1374	0.1398	0.1630	0.1457	0.1647	0.1665	0.1373	0.1448	0.1202	

## Data Availability

DNA data generated in this study are available in Genbank under accession numbers (Table S1). Other public DNA data downloaded from BOLD and Genbank are available under corresponding accession numbers (Table S1).

## Supplementary Materials

The following supporting information can be downloaded at https://www.mdpi.com/article/10.3390/insects16030301/s1. Table S1: Samples information: specimen label, taxon name, sample collection locality, and GenBank accession numbers; Table S2: Estimates of evolutionary divergence between sequences (based on *p*-distance model); Table S3: Estimates of evolutionary divergence between sequences (based on K2P model). References [18,34].

## Abbreviations

The following abbreviations are used in this manuscript:

AL	abdomen length;
ALE	anterior lateral eye;
ALS	anterior lateral spinneret;
AME	anterior median eye;
AW	abdomen width;
CL	carapace length;
CW	carapace width;
DFS	distance between the fourth pair of sigilla;
FPC	distance between the fovea and the posterior end of the carapace;
MOA	median ocular area;
PLE	posterior lateral eye;
PLS	posterior lateral spinneret;
PME	posterior median eye;
PMS	posterior median spinneret;
R1	the ratio of FPC to CL;
R2	the ratio of DFS to WFS;
TL	total length;
WFS	width of the fourth sigilla.
