# Supplementary material for: New Species of the Purse-Web Spider Genus Atypus Latreille, 1804 from Southern China (Araneae, Atypidae), with the General Natural History of Atypus Spiders
Source: Insects. 2025 Mar 13;16(3):301. doi: 10.3390/insects16030301 (PMC11942752; doi:10.3390/insects16030301)
Supplement: Supplementary file 1 [file insects-16-00301-s001.zip › insects-3483864-supplementary.pdf]

Electronic Supplementary Materials:

New Species of the Purse-Web Spider Genus *Atypus* Latreille, 1804 from Southern China (Araneae, Atypidae), with the General Natural History of *Atypus* Species

Yecheng Wu, Yang Liu, Zongguang Huang, Haiqiang Yin * and Xiang Xu *
College of Life Science, Hunan Normal University, Changsha 410081, China
* Correspondence: yinhaiqiang@hunnu.edu.cn (H.Y.); xux@hunnu.edu.cn (X.X.)

Table S1. Samples information: specimen label, taxon name, sample collection locality, and GenBank accession numbers

Species	Specimen code	GenBank accession number	Collection localities	Reference	Dataset
*Atypus baotianmanensis**	XUXI-2012-006	KP208881	Dengfeng City, Henan Province, China	[1]	I
*Atypus baotingensis**	HN-2017-032	MH279555	Baoting Country, Hainan Province, China	[2]	I
*Atypus baotingensis**	HN-2017-033	MH279556	Baoting Country, Hainan Province, China	[2]	I
*Atypus baotingensis**	HN-2017-036	MH279557	Baoting Country, Hainan Province, China	[2]	I
*Atypus baotingensis**	HN-2017-037	MH279558	Baoting Country, Hainan Province, China	[2]	I
*Atypus baotingensis**	HN-2017-037A	MH279559	Baoting Country, Hainan Province, China	[2]	I
*Atypus heterothecus**	XUXI-2012-004	KP208882	Shenzhen City, Guangdong Province, China	[1]	I
*Atypus jianfengensis**	HN-2017-003	MH279550	Ledong Country, Hainan Province, China	[2]	I
*Atypus jianfengensis**	HN-2017-005	MH279551	Ledong Country, Hainan Province, China	[2]	I
*Atypus jianfengensis**	HN-2017-007	MH279552	Ledong Country, Hainan Province, China	[2]	I
*Atypus jianfengensis**	HN-2017-008	MH279553	Ledong Country, Hainan Province, China	[2]	I
*Atypus jianfengensis**	HN-2017-010	MH279554	Ledong Country, Hainan Province, China	[2]	I
*Atypus ledongensis**	LD-001	MH279560	Ledong Country, Hainan Province, China	[2]	I
*Atypus sacculatus**	NZDZ	OR950721	Yingjiang County, Yunnan Province, China	Direct submission by Zhang,M.Y.	I
Atypus siyiensis sp. nov.	HNU1352	PV179254	ChengDu City, Sichuan Province, China	This study	I, II
Atypus siyiensis sp. nov.	HNU1353	PV179255	ChengDu City, Sichuan Province, China	This study	I, II
Atypus siyiensis sp. nov.	HNU1354	PV179256	ChengDu City, Sichuan Province, China	This study	I, II
Atypus siyiensis sp. nov.	HNU1355	PV179257	ChengDu City, Sichuan Province, China	This study	I, II
Atypus siyiensis sp. nov.	HNU1356	PV179258	ChengDu City, Sichuan Province, China	This study	I, II
Atypus siyiensis sp. nov.	HNU1357	PV179259	ChengDu City, Sichuan Province, China	This study	I, II
Atypus siyiensis sp. nov.	HNU1358	PV179260	ChengDu City, Sichuan Province, China	This study	I, II
*Atypus yajuni**	A-YN-001	KP208887	Anhui Province, Huangshan City, China	[1]	I
*Atypus yajuni**	A-YN-003	KP208886	Anhui Province, Huangshan City, China	[1]	I
Atypus yanjingensis sp. nov.	HNU1361	PV179261	Xiangxi Tujia and Miao Autonomous Prefecture, Hunan Province, China	This study	I, II
Atypus yanjingensis sp. nov.	HNU1362	PV179262	Xiangxi Tujia and Miao Autonomous Prefecture, Hunan Province, China	This study	I, II

Species	Specimen code	GenBank accession number	Collection localities	Reference	Dataset
Atypus yanjingensis sp. nov.	HNU1363	PV179263	Xiangxi Tujia and Miao Autonomous Prefecture, Hunan Province, China	This study	I, II
Atypus yanjingensis sp. nov.	HNU1364	PV179264	Xiangxi Tujia and Miao Autonomous Prefecture, Hunan Province, China	This study	I, II
Atypus yanjingensis sp. nov.	HNU1365	PV179265	Xiangxi Tujia and Miao Autonomous Prefecture, Hunan Province, China	This study	I, II
Atypus yanjingensis sp. nov.	HNU1366	PV179266	Xiangxi Tujia and Miao Autonomous Prefecture, Hunan Province, China	This study	I, II
Atypus yanjingensis sp. nov.	HNU1367	PV179267	Xiangxi Tujia and Miao Autonomous Prefecture, Hunan Province, China	This study	I, II
Atypus yaozu sp. nov.	HNU1359	PV179268	Chenzhou City, Hunan Province, China	This study	I, II
Atypus yaozu sp. nov.	HNU1360	PV179269	Chenzhou City, Hunan Province, China	This study	I, II

* sequences from GenBank

Table S2. Estimates of evolutionary divergence between sequences (based on *p*-distance model)

	1	2	3	4	5	6	7	8	9	10	11	12	13	14	15	16	17	18	19	20	21	22	23	24	25	26	27	28	29	30	31	32
1. *Atypus baotianmanensis* voucher XUXI-2012-006		0.0127	0.0127	0.0127	0.0126	0.0126	0.0148	0.0128	0.0129	0.0129	0.0129	0.0129	0.0134	0.0130	0.0132	0.0135	0.0131	0.0133	0.0133	0.0130	0.0133	0.0137	0.0135	0.0128	0.0129	0.0133	0.0134	0.0132	0.0131	0.0132	0.0132	0.0132
2. *Atypus baotingensis* voucher HN-2017-032	0.1161		0.0000	0.0000	0.0061	0.0061	0.0104	0.0131	0.0133	0.0132	0.0132	0.0131	0.0137	0.0137	0.0133	0.0135	0.0136	0.0134	0.0134	0.0138	0.0138	0.0147	0.0137	0.0137	0.0132	0.0131	0.0131	0.0130	0.0129	0.0131	0.0129	0.0132
3. *Atypus baotingensis* voucher HN-2017-036	0.1161	0.0000		0.0000	0.0061	0.0061	0.0104	0.0131	0.0133	0.0132	0.0132	0.0131	0.0137	0.0137	0.0133	0.0135	0.0136	0.0134	0.0134	0.0138	0.0138	0.0147	0.0137	0.0137	0.0132	0.0131	0.0131	0.0130	0.0129	0.0131	0.0129	0.0132
4. *Atypus baotingensis* voucher HN-2017-037A	0.1161	0.0000	0.0000		0.0061	0.0061	0.0104	0.0131	0.0133	0.0132	0.0132	0.0131	0.0137	0.0137	0.0133	0.0135	0.0136	0.0134	0.0134	0.0138	0.0138	0.0147	0.0137	0.0137	0.0132	0.0131	0.0131	0.0130	0.0129	0.0131	0.0129	0.0132
5. *Atypus baotingensis* voucher HN-2017-033	0.1161	0.0235	0.0235	0.0235		0.0000	0.0104	0.0129	0.0130	0.0129	0.0130	0.0129	0.0135	0.0135	0.0132	0.0134	0.0134	0.0134	0.0134	0.0136	0.0138	0.0144	0.0135	0.0136	0.0131	0.0127	0.0127	0.0127	0.0126	0.0128	0.0126	0.0129
6. *Atypus baotingensis* voucher HN-2017-037	0.1161	0.0235	0.0235	0.0235	0.0000		0.0104	0.0129	0.0130	0.0129	0.0130	0.0129	0.0135	0.0135	0.0132	0.0134	0.0134	0.0134	0.0134	0.0136	0.0138	0.0144	0.0135	0.0136	0.0131	0.0127	0.0127	0.0127	0.0126	0.0128	0.0126	0.0129
7. *Atypus ledongensis* voucher LD-001	0.1466	0.0677	0.0677	0.0677	0.0693	0.0693		0.0142	0.0143	0.0144	0.0143	0.0143	0.0141	0.0146	0.0148	0.0150	0.0148	0.0147	0.0147	0.0150	0.0153	0.0167	0.0153	0.0158	0.0147	0.0143	0.0144	0.0142	0.0142	0.0143	0.0142	0.0146
8. *Atypus yanjingensis* sp. nov. HNU1361	0.1238	0.1357	0.1357	0.1357	0.1313	0.1313	0.1393		0.0000	0.0021	0.0026	0.0029	0.0033	0.0027	0.0132	0.0133	0.0131	0.0130	0.0131	0.0132	0.0132	0.0140	0.0135	0.0130	0.0140	0.0134	0.0135	0.0132	0.0130	0.0133	0.0131	0.0134
9. *Atypus yanjingensis* sp. nov. HNU1366	0.1244	0.1373	0.1373	0.1373	0.1328	0.1328	0.1400	0.0000		0.0021	0.0026	0.0029	0.0033	0.0027	0.0134	0.0134	0.0132	0.0132	0.0132	0.0132	0.0132	0.0141	0.0136	0.0131	0.0140	0.0135	0.0135	0.0132	0.0131	0.0133	0.0131	0.0134
10. *Atypus yanjingensis* sp. nov. HNU1364	0.1223	0.1348	0.1348	0.1348	0.1304	0.1304	0.1417	0.0029	0.0030		0.0015	0.0021	0.0023	0.0016	0.0131	0.0132	0.0130	0.0129	0.0129	0.0134	0.0135	0.0140	0.0135	0.0130	0.0140	0.0135	0.0135	0.0132	0.0130	0.0133	0.0131	0.0134
11. *Atypus yanjingensis* sp. nov. HNU1365	0.1211	0.1347	0.1347	0.1347	0.1302	0.1302	0.1407	0.0045	0.0045	0.0015		0.0014	0.0016	0.0022	0.0132	0.0132	0.0131	0.0130	0.0130	0.0135	0.0136	0.0142	0.0137	0.0132	0.0139	0.0136	0.0136	0.0133	0.0132	0.0134	0.0132	0.0135
12. *Atypus yanjingensis* sp. nov. HNU1367	0.1229	0.1322	0.1322	0.1322	0.1278	0.1278	0.1376	0.0059	0.0060	0.0030	0.0015		0.0022	0.0027	0.0131	0.0131	0.0130	0.0129	0.0129	0.0134	0.0134	0.0143	0.0137	0.0132	0.0139	0.0135	0.0135	0.0132	0.0131	0.0134	0.0131	0.0135

	1	2	3	4	5	6	7	8	9	10	11	12	13	14	15	16	17	18	19	20	21	22	23	24	25	26	27	28	29	30	31	32
13. *Atypus yanjingensis* sp. nov. HNU1362	0.1248	0.1378	0.1378	0.1378	0.1330	0.1330	0.1374	0.0064	0.0065	0.0032	0.0016	0.0032		0.0029	0.0137	0.0137	0.0135	0.0134	0.0135	0.0140	0.0141	0.0150	0.0143	0.0137	0.0142	0.0139	0.0139	0.0137	0.0136	0.0137	0.0136	0.0140
14. *Atypus yanjingensis* sp. nov. HNU1363	0.1228	0.1395	0.1395	0.1395	0.1364	0.1364	0.1441	0.0047	0.0047	0.0016	0.0031	0.0047	0.0050		0.0134	0.0134	0.0133	0.0131	0.0131	0.0140	0.0140	0.0143	0.0138	0.0133	0.0141	0.0140	0.0140	0.0138	0.0136	0.0137	0.0137	0.0137
15. *Atypus jianfengensis* voucher HN-2017-003	0.1161	0.1366	0.1366	0.1366	0.1380	0.1380	0.1518	0.1401	0.1418	0.1378	0.1407	0.1412	0.1426	0.1379		0.0035	0.0040	0.0040	0.0044	0.0121	0.0117	0.0139	0.0134	0.0132	0.0135	0.0137	0.0138	0.0138	0.0137	0.0141	0.0137	0.0141
16. *Atypus jianfengensis* voucher HN-2017-007	0.1161	0.1366	0.1366	0.1366	0.1410	0.1410	0.1568	0.1416	0.1433	0.1393	0.1422	0.1426	0.1442	0.1395	0.0088		0.0040	0.0041	0.0045	0.0121	0.0117	0.0141	0.0133	0.0131	0.0138	0.0141	0.0142	0.0141	0.0140	0.0144	0.0140	0.0145
17. *Atypus jianfengensis* voucher HN-2017-005	0.1115	0.1395	0.1395	0.1395	0.1424	0.1424	0.1535	0.1386	0.1403	0.1363	0.1392	0.1397	0.1410	0.1364	0.0117	0.0117		0.0030	0.0037	0.0123	0.0119	0.0141	0.0133	0.0132	0.0137	0.0138	0.0139	0.0138	0.0138	0.0141	0.0137	0.0142
18. *Atypus jianfengensis* voucher HN-2017-008	0.1146	0.1366	0.1366	0.1366	0.1410	0.1410	0.1518	0.1357	0.1373	0.1333	0.1362	0.1367	0.1394	0.1332	0.0117	0.0117	0.0059		0.0020	0.0121	0.0117	0.0139	0.0132	0.0131	0.0137	0.0137	0.0138	0.0137	0.0137	0.0141	0.0137	0.0141
19. *Atypus jianfengensis* voucher HN-2017-010	0.1146	0.1351	0.1351	0.1351	0.1395	0.1395	0.1502	0.1372	0.1388	0.1348	0.1377	0.1382	0.1410	0.1348	0.0147	0.0147	0.0088	0.0029		0.0121	0.0117	0.0139	0.0133	0.0131	0.0138	0.0137	0.0139	0.0138	0.0137	0.0141	0.0137	0.0142
20. *Atypus yaozu* sp. nov. HNU1359	0.1215	0.1503	0.1503	0.1503	0.1473	0.1473	0.1675	0.1384	0.1390	0.1390	0.1411	0.1414	0.1429	0.1442	0.1086	0.1071	0.1101	0.1071	0.1057		0.0037	0.0142	0.0130	0.0131	0.0131	0.0132	0.0132	0.0132	0.0132	0.0134	0.0132	0.0135
21. *Atypus yaozu* sp. nov. HNU1360	0.1246	0.1473	0.1473	0.1473	0.1458	0.1458	0.1692	0.1384	0.1390	0.1390	0.1411	0.1414	0.1429	0.1442	0.1027	0.1012	0.1042	0.1012	0.0997	0.0089		0.0139	0.0129	0.0130	0.0132	0.0134	0.0135	0.0134	0.0134	0.0137	0.0134	0.0138
22. *Atypus sacculatus* voucher NZDZ	0.1137	0.1292	0.1292	0.1292	0.1274	0.1274	0.1571	0.1250	0.1232	0.1232	0.1230	0.1250	0.1275	0.1232	0.1239	0.1239	0.1257	0.1239	0.1239	0.1181	0.1127		0.0082	0.0066	0.0144	0.0141	0.0141	0.0141	0.0140	0.0142	0.0140	0.0141
23. *Atypus yajuni* voucher A-YN-001	0.1269	0.1331	0.1331	0.1331	0.1347	0.1347	0.1623	0.1269	0.1275	0.1254	0.1273	0.1291	0.1315	0.1291	0.1300	0.1285	0.1285	0.1269	0.1285	0.1215	0.1199	0.0409		0.0079	0.0136	0.0136	0.0137	0.0135	0.0135	0.0137	0.0135	0.0135
24. *Atypus yajuni* voucher A-YN-003	0.1146	0.1316	0.1316	0.1316	0.1300	0.1300	0.1640	0.1192	0.1198	0.1176	0.1196	0.1213	0.1214	0.1213	0.1254	0.1238	0.1269	0.1238	0.1254	0.1199	0.1168	0.0249	0.0402		0.0135	0.0138	0.0138	0.0136	0.0135	0.0136	0.0136	0.0136
25. *Atypus heterothecus voucher* XUXI-2012-004	0.1316	0.1285	0.1285	0.1285	0.1300	0.1300	0.1571	0.1517	0.1524	0.1502	0.1491	0.1509	0.1484	0.1512	0.1409	0.1424	0.1424	0.1424	0.1440	0.1340	0.1355	0.1385	0.1455	0.1378		0.0125	0.0126	0.0126	0.0124	0.0126	0.0124	0.0124
26. *Atypus siyiensis* sp. nov. HNU1352	0.1271	0.1254	0.1254	0.1254	0.1254	0.1254	0.1419	0.1333	0.1343	0.1324	0.1347	0.1322	0.1330	0.1395	0.1460	0.1490	0.1445	0.1416	0.1431	0.1414	0.1458	0.1263	0.1287	0.1333	0.1116		0.0016	0.0044	0.0041	0.0045	0.0044	0.0046

	1	2	3	4	5	6	7	8	9	10	11	12	13	14	15	16	17	18	19	20	21	22	23	24	25	26	27	28	29	30	31	32
27. *Atypus siyiensis* sp. nov. HNU1355	0.1277	0.1280	0.1280	0.1280	0.1250	0.1250	0.1443	0.1339	0.1345	0.1330	0.1351	0.1324	0.1332	0.1395	0.1488	0.1518	0.1473	0.1443	0.1458	0.1429	0.1473	0.1270	0.1308	0.1355	0.1137	0.0015		0.0041	0.0039	0.0042	0.0042	0.0044
28. *Atypus siyiensis* sp. nov. HNU1353	0.1231	0.1280	0.1280	0.1280	0.1250	0.1250	0.1443	0.1295	0.1300	0.1286	0.1306	0.1280	0.1284	0.1348	0.1473	0.1503	0.1458	0.1429	0.1443	0.1458	0.1503	0.1252	0.1277	0.1324	0.1106	0.0134	0.0119		0.0015	0.0022	0.0021	0.0023
29. *Atypus siyiensis* sp. nov. HNU1356	0.1223	0.1268	0.1268	0.1268	0.1239	0.1239	0.1443	0.1268	0.1284	0.1259	0.1287	0.1263	0.1268	0.1332	0.1460	0.1490	0.1445	0.1416	0.1431	0.1458	0.1503	0.1239	0.1269	0.1300	0.1084	0.0119	0.0104	0.0015		0.0015	0.0015	0.0016
30. *Atypus siyiensis* sp. nov. HNU1357	0.1254	0.1309	0.1309	0.1309	0.1279	0.1279	0.1467	0.1309	0.1309	0.1294	0.1315	0.1294	0.1290	0.1343	0.1522	0.1553	0.1507	0.1476	0.1492	0.1507	0.1553	0.1261	0.1302	0.1333	0.1111	0.0137	0.0122	0.0030	0.0015		0.0022	0.0023
31. *Atypus siyiensis* sp. nov. HNU1358	0.1240	0.1283	0.1283	0.1283	0.1254	0.1254	0.1452	0.1289	0.1299	0.1280	0.1302	0.1278	0.1282	0.1348	0.1460	0.1490	0.1445	0.1431	0.1445	0.1458	0.1503	0.1228	0.1287	0.1318	0.1085	0.0133	0.0119	0.0030	0.0015	0.0030		0.0000
32. *Atypus siyiensis* sp. nov. HNU1354	0.1240	0.1272	0.1272	0.1272	0.1256	0.1256	0.1459	0.1319	0.1319	0.1303	0.1319	0.1303	0.1307	0.1331	0.1476	0.1507	0.1460	0.1444	0.1460	0.1460	0.1507	0.1239	0.1287	0.1319	0.1083	0.0141	0.0126	0.0031	0.0016	0.0032	0.0000	

Table S3. Estimates of evolutionary divergence between sequences (based on K2P model)

	1	2	3	4	5	6	7	8	9	10	11	12	13	14	15	16	17	18	19	20	21	22	23	24	25	26	27	28	29	30	31	32
1. *Atypus baotianmanensis* voucher XUXI-2012-006		0.0153	0.0153	0.0153	0.0151	0.0151	0.0191	0.0160	0.0161	0.0157	0.0156	0.0158	0.0166	0.0158	0.0154	0.0153	0.0150	0.0152	0.0151	0.0155	0.0157	0.0162	0.0160	0.0149	0.0164	0.0159	0.0160	0.0155	0.0155	0.0159	0.0157	0.0158
2. *Atypus baotingensis* voucher HN-2017-032	0.1275		0.0000	0.0000	0.0060	0.0060	0.0112	0.0166	0.0168	0.0164	0.0165	0.0162	0.0172	0.0171	0.0163	0.0164	0.0167	0.0164	0.0161	0.0177	0.0173	0.0169	0.0163	0.0160	0.0162	0.0155	0.0158	0.0159	0.0157	0.0163	0.0158	0.0160
3. *Atypus baotingensis* voucher HN-2017-036	0.1275	0.0000		0.0000	0.0060	0.0060	0.0112	0.0166	0.0168	0.0164	0.0165	0.0162	0.0172	0.0171	0.0163	0.0164	0.0167	0.0164	0.0161	0.0177	0.0173	0.0169	0.0163	0.0160	0.0162	0.0155	0.0158	0.0159	0.0157	0.0163	0.0158	0.0160
4. *Atypus baotingensis* voucher HN-2017-037A	0.1275	0.0000	0.0000		0.0060	0.0060	0.0112	0.0166	0.0168	0.0164	0.0165	0.0162	0.0172	0.0171	0.0163	0.0164	0.0167	0.0164	0.0161	0.0177	0.0173	0.0169	0.0163	0.0160	0.0162	0.0155	0.0158	0.0159	0.0157	0.0163	0.0158	0.0160
5. *Atypus baotingensis* voucher HN-2017-033	0.1278	0.0239	0.0239	0.0239		0.0000	0.0117	0.0161	0.0163	0.0160	0.0160	0.0157	0.0167	0.0169	0.0163	0.0167	0.0169	0.0167	0.0165	0.0176	0.0173	0.0171	0.0168	0.0161	0.0166	0.0159	0.0159	0.0159	0.0158	0.0163	0.0158	0.0164
6. *Atypus baotingensis* voucher HN-2017-037	0.1278	0.0239	0.0239	0.0239	0.0000		0.0117	0.0161	0.0163	0.0160	0.0160	0.0157	0.0167	0.0169	0.0163	0.0167	0.0169	0.0167	0.0165	0.0176	0.0173	0.0171	0.0168	0.0161	0.0166	0.0159	0.0159	0.0159	0.0158	0.0163	0.0158	0.0164
7. *Atypus ledongensis* voucher LD-001	0.1668	0.0717	0.0717	0.0717	0.0738	0.0738		0.0172	0.0173	0.0176	0.0174	0.0171	0.0170	0.0181	0.0183	0.0189	0.0190	0.0187	0.0184	0.0202	0.0202	0.0204	0.0195	0.0195	0.0204	0.0182	0.0185	0.0183	0.0183	0.0186	0.0182	0.0188
8. *Atypus yanjingensis* sp. nov. HNU1361	0.1376	0.1512	0.1512	0.1512	0.1458	0.1458	0.1557		0.0000	0.0021	0.0026	0.0029	0.0032	0.0028	0.0169	0.0171	0.0170	0.0168	0.0169	0.0166	0.0164	0.0178	0.0167	0.0158	0.0185	0.0165	0.0166	0.0165	0.0162	0.0166	0.0164	0.0171
9. *Atypus yanjingensis* sp. nov. HNU1366	0.1384	0.1532	0.1532	0.1532	0.1477	0.1477	0.1566	0.0000		0.0021	0.0026	0.0030	0.0032	0.0028	0.0172	0.0173	0.0172	0.0170	0.0172	0.0167	0.0165	0.0176	0.0168	0.0158	0.0185	0.0167	0.0167	0.0166	0.0164	0.0166	0.0166	0.0171
10. *Atypus yanjingensis* sp. nov. HNU1364	0.1357	0.1501	0.1501	0.1501	0.1446	0.1446	0.1587	0.0030	0.0030		0.0015	0.0021	0.0023	0.0016	0.0167	0.0169	0.0167	0.0165	0.0167	0.0166	0.0165	0.0176	0.0166	0.0156	0.0182	0.0164	0.0165	0.0164	0.0161	0.0165	0.0164	0.0169
11. *Atypus yanjingensis* sp. nov. HNU1365	0.1343	0.1500	0.1500	0.1500	0.1446	0.1446	0.1576	0.0045	0.0045	0.0015		0.0015	0.0016	0.0022	0.0171	0.0173	0.0172	0.0170	0.0172	0.0169	0.0168	0.0176	0.0168	0.0158	0.0180	0.0167	0.0168	0.0167	0.0164	0.0167	0.0166	0.0171
12. *Atypus yanjingensis* sp. nov. HNU1367	0.1365	0.1469	0.1469	0.1469	0.1415	0.1415	0.1537	0.0060	0.0060	0.0030	0.0015		0.0023	0.0027	0.0172	0.0174	0.0172	0.0171	0.0172	0.0170	0.0168	0.0178	0.0170	0.0160	0.0183	0.0164	0.0164	0.0163	0.0161	0.0164	0.0163	0.0169

	1	2	3	4	5	6	7	8	9	10	11	12	13	14	15	16	17	18	19	20	21	22	23	24	25	26	27	28	29	30	31	32
13. *Atypus yanjingensis* sp. nov. HNU1362	0.1392	0.1538	0.1538	0.1538	0.1479	0.1479	0.1534	0.0065	0.0065	0.0032	0.0016	0.0032		0.0030	0.0177	0.0179	0.0178	0.0177	0.0179	0.0176	0.0174	0.0189	0.0179	0.0167	0.0186	0.0170	0.0170	0.0168	0.0167	0.0169	0.0168	0.0175
14. *Atypus yanjingensis* sp. nov. HNU1363	0.1363	0.1561	0.1561	0.1561	0.1523	0.1523	0.1620	0.0047	0.0047	0.0016	0.0031	0.0047	0.0051		0.0173	0.0175	0.0173	0.0171	0.0172	0.0174	0.0173	0.0177	0.0171	0.0161	0.0184	0.0175	0.0175	0.0174	0.0172	0.0173	0.0174	0.0173
15. *Atypus jianfengensis* voucher HN-2017-003	0.1269	0.1513	0.1513	0.1513	0.1535	0.1535	0.1708	0.1573	0.1594	0.1542	0.1579	0.1585	0.1605	0.1546		0.0036	0.0042	0.0042	0.0048	0.0152	0.0146	0.0171	0.0162	0.0165	0.0174	0.0171	0.0173	0.0172	0.0170	0.0178	0.0170	0.0177
16. *Atypus jianfengensis* voucher HN-2017-007	0.1270	0.1514	0.1514	0.1514	0.1573	0.1573	0.1774	0.1591	0.1612	0.1560	0.1597	0.1603	0.1624	0.1565	0.0089		0.0042	0.0042	0.0048	0.0150	0.0144	0.0169	0.0160	0.0162	0.0175	0.0172	0.0174	0.0173	0.0171	0.0179	0.0171	0.0178
17. *Atypus jianfengensis* voucher HN-2017-005	0.1213	0.1550	0.1550	0.1550	0.1591	0.1591	0.1730	0.1554	0.1575	0.1523	0.1559	0.1565	0.1584	0.1526	0.0119	0.0119		0.0030	0.0036	0.0150	0.0144	0.0168	0.0161	0.0163	0.0173	0.0171	0.0173	0.0173	0.0171	0.0179	0.0171	0.0178
18. *Atypus jianfengensis* voucher HN-2017-008	0.1250	0.1513	0.1513	0.1513	0.1572	0.1572	0.1709	0.1518	0.1539	0.1488	0.1523	0.1530	0.1565	0.1488	0.0119	0.0118	0.0059		0.0021	0.0148	0.0142	0.0167	0.0158	0.0161	0.0174	0.0168	0.0170	0.0169	0.0167	0.0175	0.0169	0.0175
19. *Atypus jianfengensis* voucher HN-2017-010	0.1250	0.1494	0.1494	0.1494	0.1553	0.1553	0.1688	0.1538	0.1558	0.1507	0.1543	0.1549	0.1585	0.1509	0.0149	0.0149	0.0089	0.0029		0.0146	0.0140	0.0167	0.0159	0.0164	0.0176	0.0170	0.0172	0.0171	0.0169	0.0177	0.0171	0.0177
20. *Atypus yaozu* sp. nov. HNU1359	0.1341	0.1690	0.1690	0.1690	0.1656	0.1656	0.1920	0.1548	0.1555	0.1555	0.1581	0.1585	0.1605	0.1623	0.1192	0.1175	0.1210	0.1173	0.1155		0.0037	0.0167	0.0158	0.0162	0.0172	0.0166	0.0167	0.0169	0.0169	0.0176	0.0169	0.0173
21. *Atypus yaozu* sp. nov. HNU1360	0.1379	0.1650	0.1650	0.1650	0.1635	0.1635	0.1941	0.1546	0.1554	0.1554	0.1580	0.1583	0.1603	0.1622	0.1120	0.1103	0.1138	0.1102	0.1084	0.0090		0.0161	0.0155	0.0158	0.0172	0.0168	0.0170	0.0172	0.0172	0.0179	0.0172	0.0177
22. *Atypus sacculatus* voucher NZDZ	0.1238	0.1422	0.1422	0.1422	0.1402	0.1402	0.1778	0.1374	0.1353	0.1352	0.1349	0.1374	0.1403	0.1352	0.1362	0.1363	0.1384	0.1361	0.1361	0.1297	0.1232		0.0090	0.0069	0.0176	0.0169	0.0170	0.0166	0.0165	0.0169	0.0164	0.0165
23. *Atypus yajuni* voucher A-YN-001	0.1404	0.1473	0.1473	0.1473	0.1495	0.1495	0.1848	0.1401	0.1408	0.1381	0.1404	0.1426	0.1456	0.1427	0.1448	0.1430	0.1428	0.1409	0.1428	0.1344	0.1326	0.0424		0.0082	0.0173	0.0156	0.0158	0.0156	0.0155	0.0159	0.0157	0.0158
24. *Atypus yajuni* voucher A-YN-003	0.1253	0.1455	0.1455	0.1455	0.1439	0.1439	0.1879	0.1307	0.1314	0.1288	0.1311	0.1332	0.1334	0.1332	0.1385	0.1367	0.1405	0.1366	0.1385	0.1323	0.1283	0.0254	0.0417		0.0168	0.0159	0.0160	0.0159	0.0157	0.0161	0.0159	0.0160
25. *Atypus heterothecus* voucher XUXI-2012-004	0.1457	0.1418	0.1418	0.1418	0.1434	0.1434	0.1780	0.1712	0.1722	0.1692	0.1679	0.1702	0.1670	0.1705	0.1569	0.1587	0.1588	0.1587	0.1606	0.1488	0.1506	0.1539	0.1630	0.1532		0.0143	0.0145	0.0144	0.0142	0.0147	0.0142	0.0143
26. *Atypus siyiensis* sp. nov. HNU1352	0.1404	0.1382	0.1382	0.1382	0.1380	0.1380	0.1596	0.1484	0.1496	0.1473	0.1500	0.1469	0.1478	0.1561	0.1637	0.1674	0.1618	0.1582	0.1601	0.1583	0.1640	0.1389	0.1421	0.1481	0.1217		0.0015	0.0046	0.0044	0.0048	0.0046	0.0049

	1	2	3	4	5	6	7	8	9	10	11	12	13	14	15	16	17	18	19	20	21	22	23	24	25	26	27	28	29	30	31	32
27. *Atypus siyiensis* sp. nov. HNU1355	0.1411	0.1414	0.1414	0.1414	0.1375	0.1375	0.1626	0.1491	0.1499	0.1480	0.1505	0.1471	0.1481	0.1561	0.1673	0.1711	0.1653	0.1617	0.1636	0.1603	0.1660	0.1398	0.1448	0.1509	0.1243	0.0015		0.0043	0.0040	0.0044	0.0043	0.0045
28. *Atypus siyiensis* sp. nov. HNU1353	0.1351	0.1412	0.1412	0.1412	0.1373	0.1373	0.1623	0.1435	0.1442	0.1423	0.1448	0.1415	0.1420	0.1500	0.1653	0.1691	0.1634	0.1598	0.1617	0.1639	0.1696	0.1375	0.1409	0.1469	0.1205	0.0135	0.0120		0.0016	0.0022	0.0022	0.0023
29. *Atypus siyiensis* sp. nov. HNU1356	0.1343	0.1399	0.1399	0.1399	0.1361	0.1361	0.1625	0.1403	0.1422	0.1392	0.1425	0.1395	0.1401	0.1482	0.1638	0.1675	0.1619	0.1583	0.1602	0.1640	0.1697	0.1359	0.1401	0.1440	0.1179	0.0120	0.0105	0.0015		0.0015	0.0016	0.0017
30. *Atypus siyiensis* sp. nov. HNU1357	0.1381	0.1449	0.1449	0.1449	0.1409	0.1409	0.1656	0.1453	0.1453	0.1434	0.1459	0.1433	0.1429	0.1495	0.1717	0.1756	0.1697	0.1660	0.1680	0.1703	0.1762	0.1386	0.1440	0.1481	0.1211	0.0139	0.0123	0.0031	0.0015		0.0022	0.0023
31. *Atypus siyiensis* sp. nov. HNU1358	0.1364	0.1418	0.1418	0.1418	0.1379	0.1379	0.1637	0.1429	0.1441	0.1417	0.1444	0.1414	0.1418	0.1502	0.1638	0.1675	0.1619	0.1602	0.1621	0.1640	0.1697	0.1346	0.1422	0.1463	0.1181	0.0134	0.0120	0.0030	0.0015	0.0031		0.0000
32. *Atypus siyiensis* sp. nov. HNU1354	0.1364	0.1403	0.1403	0.1403	0.1381	0.1381	0.1646	0.1466	0.1466	0.1446	0.1464	0.1444	0.1449	0.1481	0.1659	0.1699	0.1638	0.1621	0.1641	0.1642	0.1703	0.1359	0.1422	0.1463	0.1178	0.0143	0.0127	0.0031	0.0016	0.0032	0.0000	

Reference:

1. Xu, X.; Liu, F.; Cheng, R.; Chen, J.; Xu, X.; Zhang, Z.; Ono, H.; Pham, D.S.; Norma-Rashid, Y.; Arnedo, M.A.; et al. Extant Primitively Segmented Spiders Have Recently Diversified from an Ancient Lineage. *P. R. Soc. B.* **2015**, *282*, 20142486, doi:10.1098/rspb.2014.2486.
2. Li, F.; Xu, X.; Zhang, Z.; Liu, F.; Zhang, H.; Li, D. Two New Species of the Purse-Web Spider Genus *Atypus* Latreille, 1804 from Hainan Island, China (Araneae, Atypidae). *Zookeys* **2018**, *762*, 47–57, doi:10.3897/zookeys.762.23282.
